# Genetic dissection of formate hydrogenlyase-2 function: a metalloenzyme involved in bacterial hydrogen production

**DOI:** 10.1007/s00775-026-02168-7

**Published:** 2026-07-23

**Authors:** Thomas C.P. Reed, Alexander J. Finney, Magali Roger, Frank Sargent

**Affiliations:** 1 Newcastle University Biosciences Institute, Newcastle University, Newcastle upon Tyne, NE2 4HH England, UK; 2 Present Address: Institut de Microbiologie de la Méditerranée, 31 Chemin Joseph Aiguier, Marseille Cedex 09, 70071, 13402 CS France; 3 Present Address: SOLASTA Bio Ltd, St. Vincent Plaza, 319 St. Vincent Street, Glasgow, G2 5RZ Scotland, UK

**Keywords:** Mixed acid fermentation, Anaerobic metabolism, Molecular genetics, Hydrogenase, Formate dehydrogenase, Formate hydrogenlyase

## Abstract

Formate hydrogenlyases are metalloenzymes that can either produce molecular hydrogen gas or use H_2_ to convert carbon dioxide to formic acid, thus potentially contributing doubly to a sustainable energy future. The structure of formate hydrogenlyase reveals a membrane-bound redox enzyme that shares a common ancestor with the mitochondrial complex I (NADH dehydrogenase). As such, formate hydrogenlyase falls into a category of so‐called ‘complex‐I‐like’ enzymes, that are found in bacteria, archaea and eukaryotic organelles. They share a common core structure of a membrane arm (most likely involved in proton or ion translocation) and a peripheral arm containing metal cofactors and involved in electron transfer. In this work, we clone a gene cluster from *Pectobacterium atrosepticum* encoding formate hydrogenlyase-2 (FHL-2). A bank of *Escherichia coli* host strains, themselves devoid of various combinations of native formate hydrogenlyase genes, are employed to characterise FHL-2. We demonstrate that *P. atrosepticum* FHL-2 is active in an *E. coli* host in that it can generate H_2_ under fermentative growth conditions. Unlike native *E. coli* formate hydrogenlyase-1 (FHL-1), recombinant *P. atrosepticum* FHL-2 cannot perform the reverse reaction and generate formic acid from H_2_ and CO_2_. By testing different combinations of genes by taking an in vivo cross-complementation approach we conclude that the extended membrane arm exhibited by FHL-2 is a major factor in controlling directionality of the enzyme.

**Supplementary Information:**

The online version contains supplementary material available at https://doi.org/10.1007/s00775-026-02168-7.

## Introduction

The model organism *Escherichia coli* is a facultatively anaerobic γ-Proteobacterium and therefore possesses a flexible metabolism that enables survival in a range of environmental conditions [1]. In the presence of oxygen, *E. coli* will preferentially perform aerobic respiration using short, quinone-dependent, electron transport chains for oxidative phosphorylation [2]. Under anaerobic conditions, however, *E. coli* can adapt its respiratory electron transport chain to utilise alternative terminal electron acceptors, such as nitrate, fumarate and dimethyl sulfoxide [2, 3]. In the complete absence of exogeneous terminal electron acceptors, *E. coli* will instead conduct ‘mixed-acid’ fermentation [4], where glucose is metabolised via the Embden-Meyerhof-Parnas glycolytic pathway to generate ATP by substrate level phosphorylation [5]. This process results in the excretion of ethanol, acetic acid, formic acid and lactic acid as waste products [6]. Extracellular accumulation of formic acid, and the associated pH decrease, induces the production of the formate hydrogenlyase-1 complex (FHL-1), which then functions in pH regulation by catalysing the disproportionation of formic acid to molecular hydrogen and carbon dioxide [7]. This formate-dependent H_2_ evolution reaction can be considered the physiological ‘forward’ reaction of FHL-1. However, FHL-1 is also readily reversible and can carry out the ‘reverse’ reaction as a hydrogen-dependent CO_2_ reductase (HDCR) where formic acid can be generated from gaseous substrates under permissive environmental conditions [8].

The FHL-1 complex is encoded by the *hycABCDEFGHI* operon and the distally located *fdhF* gene. Expression of both the *hyc* operon and *fdhF* is mediated by the transcriptional activator FhlA and repressor HycA [9, 10]. The functional FHL-1 complex is comprised of a membrane arm made of two integral membrane proteins, HycC and HycD, connected to a cytoplasmic, catalytic, peripheral or soluble arm. The peripheral arm is comprised of the molybdenum- and selenium-dependent formate dehydrogenase-H (FdhF) connected to a group 4 [NiFe] hydrogenase (HycEG) by two Fe-S proteins (HycB and HycF) [9, 11, 12]. The hydrogenase component is called hydrogenase-3 (‘Hyd-3’) in *E. coli*. There is sequence similarity between all of the FHL-1 subunits and subunits of the respiratory NADH dehydrogenase complex I [13]. Formate hydrogenlyase can therefore be considered a member of a large group of membrane-bound enzymes collectively termed the ‘NADH dehydrogenase-like’, or ‘complex I-like’, enzymes.

The apparent evolutionary link between FHL-1 and complex I is another membrane-bound hydrogenase termed FHL-2. The FHL-2 complex is predicted to be comprised of a peripheral arm constituting a formate dehydrogenase (FdhF) linked to a group 4 [NiFe] hydrogenase [14] (this time termed ‘Hyd-4’) via three Fe-S cluster proteins (HyfA, HyfH and HyfI). The key difference between FHL-1 and FHL-2 type proteins is that FHL-2 is predicted to comprise a larger five-subunit membrane arm, that much more closely resembles the larger membrane arm found in *bona fide* complex I and other complex-I-like proteins.

The *E. coli* genome contains a gene cluster (*hyfABCDEFGHIJR-focB*) that would encode an FHL-2 complex [15]. However, the evidence for physiological FHL-2 activity in *E. coli* K-12 is inconsistent, with some studies highlighting that the FHL-2 complex is possibly not transcribed under natural [16] or other laboratory conditions [17]. Furthermore, *E. coli* strains lacking functional FHL-1 are usually incapable of producing hydrogen gas [18], while the inactivation of FHL-2 can have little or no effect on overall hydrogen production levels [19]. Nevertheless, it should be noted that there is some data pointing to FHL-2 activity in *E. coli* [20, 21].

**Fig. 1:**
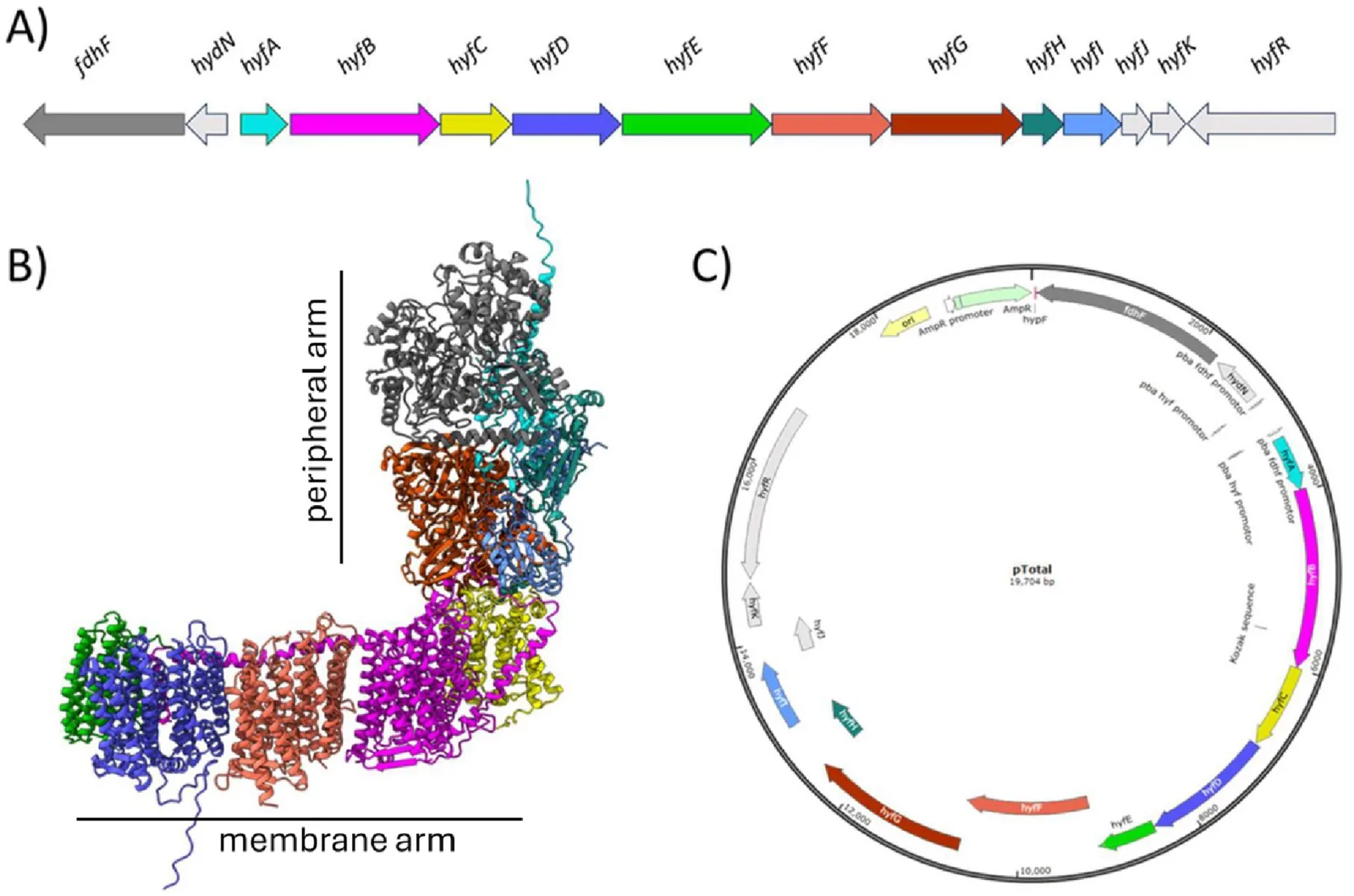
Cloning FHL-2 from *Pectobaterium atrosepticum*. **(A)** The gene cluster encoding FHL-2 from *P. atrosepticum*. **(B)** A model of the predicted structure of the *P. atrosepticum* FHL-2 complex. Subunits are colour-coded exactly as the gene names in panel (A). The membrane arm comprises five proteins – HyfB, -C, -D, -E, and -F. The peripheral arm comprises five proteins - HyfA, -G, -H, -I, and FdhF. **(C)** A map of the 19.7 kbp pTotal (Amp^R^) plasmid carrying the entire FHL-2 cluster

Although *E. coli* FHL-2 remains to be more fully understood, research based on alternative model organisms has identified functional FHL-2 complexes in *Vibrio tritonius* [22], *Trabulsiella guamensis* [23] and *Pectobacterium atrosepticum* [24]. The FHL-2 from *P. atrosepticum* is encoded by the *hyfABCDEFGHIJK-hyfR* operon adjacent to the *fdhF*-*hydN* operon with the two predicted to be transcribed from divergent promotors [24, 25]. The *hydN* gene encodes a protein very similar to HycB and HyfA (Supplementary Figures S1-S10), which are considered the small subunits of the formate dehydrogenase in FHL-1 and FHL-2, respectively. Interestingly, the FdhF formate dehydrogenase of *P. atrosepticum* FHL-2 is predicted to display a catalytic cysteine residue at its active site rather than selenocysteine, as seen in *E. coli* FdhF [24, 26]. Similar to *E. coli* FHL-1 and FHL-2, the peripheral arm of the FHL-2 complex from *P. atrosepticum* contains the catalytic large subunit of a group 4 [NiFe] hydrogenase (encoded by *hyfG*). This protein is the equivalent to HycE in FHL-1 (Supplementary Figures S1-S10). The hydrogenase large subunit is linked directly to a hydrogenase small subunit (HyfI in FHL-2 and HycG in FHL-1) and the dimer is connected to the formate dehydrogenase by another Fe-S cluster protein (HyfH in FHL-2 and HycF in FHL-1) (Fig. 1B). The membrane arm of FHL-2 is comprised of 5 integral membrane subunits: the direct membrane anchor for the peripheral arm - HyfC; three antiporter-like protein homologs (HyfB, HyfD and HyfF); and the HyfE protein, which is not found in FHL-1 but shares some sequence similarity with NuoK from complex I [24].

*P. atrosepticum* is a phytopathogenic enterobacterium known to cause soft rot diseases [25]. This, combined with its slow growth rate and preference for relatively low temperatures, make it a suboptimal host for large scale biochemical or structural studies. Therefore, in this work, the genes encoding FHL-2 from *P. atrosepticum* were characterised using a plasmid-based approach. This enabled the FHL-2 complex to be studied in an *E. coli* host, where it was shown to be capable of producing molecular hydrogen during fermentative growth. Additionally, unlike FHL-1, the recombinant FHL-2 complex appears unable to function in reverse as a hydrogen-dependent carbon dioxide reductase (HDCR). The inability of FHL-2 to function bidirectionally may be a consequence of the biochemical properties of the Hyd-4 component of FHL-2 rather than the formate dehydrogenase component.

## Materials and methods

### Strain construction

The strains used in this study are listed in Table 1. Gene deletions were conducted by homologous recombination using pMAK705 protocol from the parental strain FTD89 (MC4100 Δ*hyaB*, Δ*hybC*) [27]. FTD200, which lacks all functional hydrogenases, was constructed from FTD89 through the removal of the *hyc* and *hyf* operons. First, 500 bp up- and downstream of the *hyc* operon (*hycA-I*) were amplified by PCR and assembled in pMAK705 [27]. Following this, the *hyf* operon was deleted using pGS1039 [17]. FTD250 was constructed from FTD200 via the deletion of *fdhF* using a pMAK705 vector, constructed as previously described, to provide a ‘clean’ platform strain for the expression of non-native FHL complexes. The strain 89F was made using the same protocol to allow for the testing of non-native formate dehydrogenases in complex with Hyd-3.

**Table 1:** E. coli K-12 strains used and constructed in this study

Strain	Genotype	Reference
FTD89	As MC4100, Δ*hyaB*, Δ*hybC*	[28]
FTD147	As MC4100, Δ*hyaB*, Δ*hybC*, Δ*hycE*	[18]
FTD150	As MC4100, Δ*hyaB*, Δ*hybC*, Δ*hycE*, Δ*hyfB-R*	[19]
FTD250	As FTD89, Δ*hycA-I*, Δ*hyfB-R*, Δ*fdhF*	This Work
FTD200	As FTD89, Δ*hycA-I*, Δ*hyfB-R*	This Work
89F	As FTD89, Δ*fdhF*	This Work
MG059e1	As MG1655, *hycE*^*His*^ (internal His-tag)	[29]
MR60	As MG059e1, Δ*hycA*, Δ*fdhF*, Δ*hyaB*, Δ*hybC*, Δ*pflA*, Δ*fdhE* PT5 ɸ*fdhF*HA::*hycB*	[30]
Tom001	As MR60, Δ*hycCD*	[30]

### Plasmid construction

For the expression of FHL-2 in an *Escherichia coli* host, genes encoding FHL-2 were amplified from the genome of *P. atrosepticum* SCRI1043 using Phusion™ High Fidelity DNA polymerase (Thermo Scientific) and assembled into pT7.5/Litmus. Due to the size of the operon encoding FHL-2 it was amplified as two fragments using the internal BglII site within *hyfE*. The downstream fragment was amplified via PCR and digested using HindIII and BglII, while the upstream fragment was amplified before digestion with BglII and XbaI. The fragments were assembled into pT7.5/Litmus using the HindIII and XbaI sites. The resultant plasmid was named pTotal. Differences between the cloned sequence and that of the SCRI1043 genome sequence are listed in Supplementary Table S1.

All complementation plasmids were constructed using HiFi Assembly (NEB) using DNA amplified from the genome of *P. atrosepticum* SCRI1043 or *E. coli* MG1655. Genes were cloned into pSU-PROM which contains the constitutive *tatA* promoter [31].

### Whole-cell H_2_ production

Hydrogen production of anaerobic cultures in sealed Hungate Tubes was quantified by gas chromatography [32]. Cells were grown in LB Miller medium supplemented with 0.4% (w/v) glucose and 0.2% (w/v) formate at 37 °C for 20 h. 1 ml of headspace gas was collected using a gas-tight syringe with Luer lock valve (SGE). Samples were injected into a Shimadzu GC-2014 gas chromatograph fitted with a capillary column and TCD detector. Hydrogen was quantified using a hydrogen standard curve and normalised to culture density (OD 600 nm).

### Whole-cell formate production

Cells were grown in LB medium supplemented with 0.4% (w/v) glucose under anaerobic conditions before being harvested by centrifugation for 10 min at 2773 x *g* and 4 °C. Cells were washed thrice with 20 mmol/L 3-(N-morpholino)propanesulfonic acid (MOPS) buffer, pH 7.4, before being resuspended to an OD_600nm_ of 1.0 +/- 0.02. No anaerobic precautions were taken during these washing steps though cell resuspension was carried out gently using a glass homogeniser. Washed cells were transferred to Hungate tubes before the headspace was sparged with N_2_ followed by H_2_ and finally was subjected to the injection of 5 ml gaseous CO_2_. Cells were incubated at 37 °C without agitation for 20 h. Liquid samples were taken via syringe and clarified before HPLC analysis using a an UltiMate 3000 uHPLC system (Thermo) equipped with an Aminex HPX-87 H column (BioRad) using a RefractoMax521 refractive index detector (Thermo) and a Variable Wavelength Detector-3100 at 210 nm (Thermo). Samples of 10 µl previously clarified through a 0.45 μm filter were injected into the HPLC system that had been equilibrated with 5 mmol/L H_2_SO_4_ with a flow of 0.8 mL/min at 50 °C. Formate was quantified using a standard curve.

## Results

### Production of functional FHL-2 in *E. coli*

**Fig. 2:**
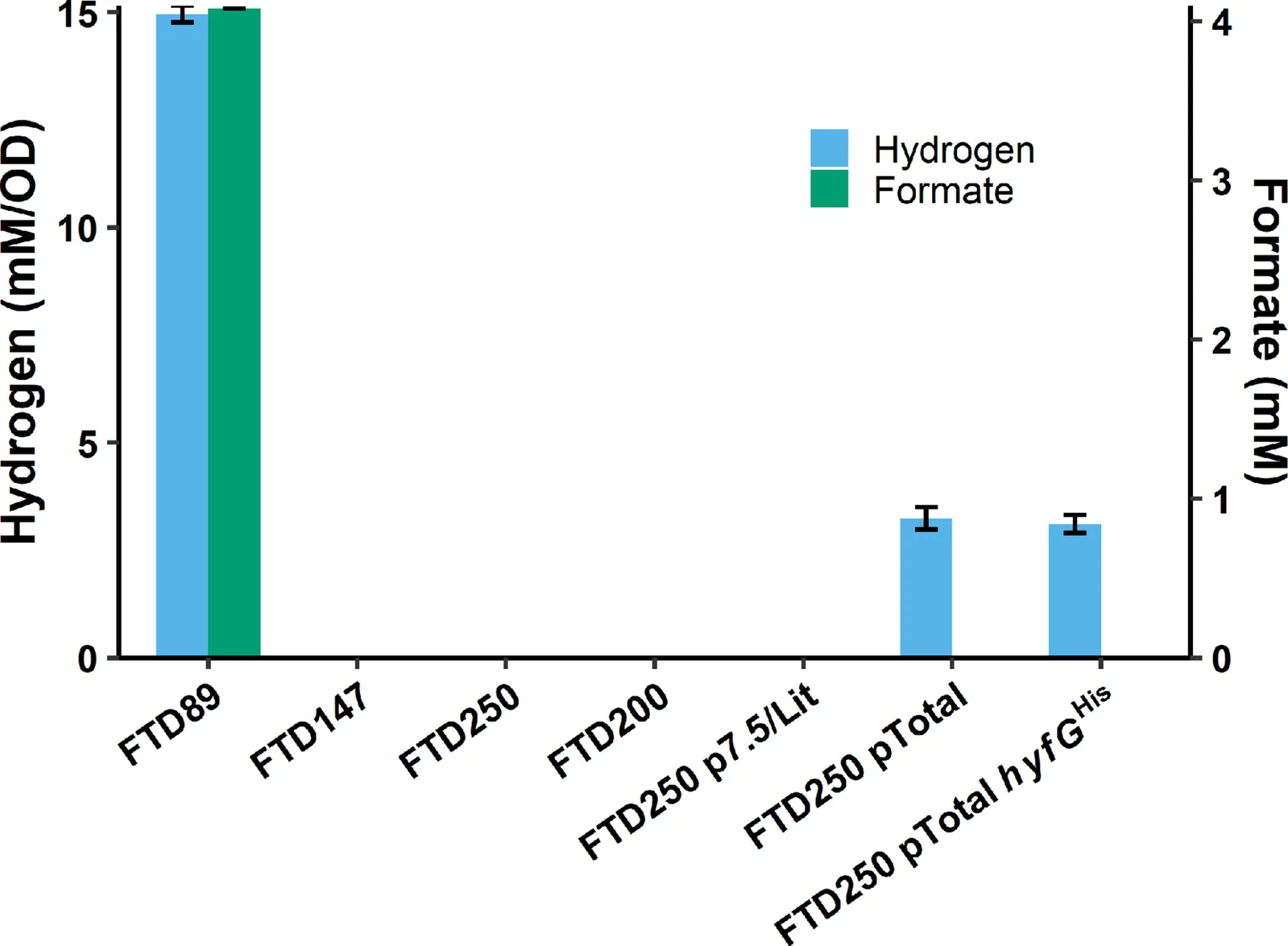
FHL-2 from *Pectobaterium atrosepticum* is capable of producing hydrogen during fermentation in an *E. coli* host. *E. coli *strains expressing either FHL-1 or FHL-2 were grown under anearobic fermentative conditions in LB medium supplimented with 0.4% (w/v) glucose and 0.2% (w/v) formate for 20 h at 37 °C (*n* = 3). To determine hydrogen production activity (forward reaction) of the FHL complexes, cultures were grown in Hungate tubes to allow for hydrogen accumulation in the headspace. Hydrogen was removed from the headspace using a gas-tight, Luer-lock syringe and quantified by gas chromatography. To determine the formate production activity (reverse reaction) of the FHL complexes, anaerobic cultures were washed three times in MOPS buffer, pH 7.4 and resuspended to an OD 600 nm of 1.0 before being transferred to Hungate tubes and supplied with gaseous substrates. Cell suspensions were incubated at 37 °C for 20 h before sampling. Formate production was quantified via HPLC. Error bars represent standard error of the mean

To enable characterisation of *P. atrospeticum* FHL-2, an *E. coli* K-12 host strain was required that was devoid of hydrogenase activity and all endogenous FHL-1 and FHL-2 structural genes. The FTD150 strain (Δ*hyaB* Δ*hybC* Δ*hycE* Δ*hyfB-R*, Table 1) was therefore used as a starting point [18]. This *E. coli* strain already carries in-frame deletions in the catalytic subunits of the three active [NiFe]-hydrogenases in *E. coli* [33] as well as a deletion of the *hyfBCDEFGHIJ-hyfR* cluster encoding Hyd-4 [17]. Note that an ‘in-frame deletion’ carefully removes all coding sequence from a gene while preserving start codon, stop codon, and any internal regulatory sequences related to adjacent genes (such as ribosome binding sites). This approach is particularly important when studying multi-cistronic operons to minimise downstream (or ‘polar’) effects on translation. To further modify this strain, first the entire *hyc* operon (*hycA-I*) was deleted from FTD150 resulting in the *E. coli* strain FTD200 (Δ*hyaB* Δ*hybC* Δ*hycA-I* Δ*hyfB-R*, Table 1). Then, the gene encoding the FdhF formate dehydrogenase was deleted from strain FTD200, resulting in the strain FTD250 (Δ*hyaB* Δ*hybC* Δ*hycA-I* Δ*hyfB-R* Δ*fdhF*, Table 1). When incubated under anaerobic, fermentative growth conditions, the new FTD250 strain was found to be incapable of biological H_2_ evolution (Fig. 2).

Next, the entire 15 kbp gene cluster encoding *P. atrosepticum* FHL-2 (i.e. the 11-cistron operon encoding the Hyd-4 module and its associated accessory proteins, *hyfA-R*, as well as the region comprising the genes encoding FdhF and HydN, Fig. 1A) were cloned into a single plasmid including the native, divergent, promotors of both operons (Fig. 1C; Table 2). Given the formate-responsive regulator HyfR would be present on this construct, and the relatedness of *P. atrospeticum* to gamma-Proteobacteria like *E. coli*, it was considered that the two operons could be anaerobically expressed in an *E. coli* host and there would be little need for synthetic overexpression strategies at this stage.

**Table 2:** Plasmids constructed in this study

Name	Relevant Genotype	Resistance
pTotal	pT7-Litmus carrying the entire *fdhF-hyfR* gene cluster for *P. atrosepticum* FHL-2	Amp
pTotal-*hyfG*^His^	As pTotal with internal 10-His coding sequence in *hyfG* between codons for Gly-93 and Glu-94	Amp
pTotal-*hyfG*^His^ Δ*hyfB-F*	As pTotal-*hyfG*^His^ with in-frame deletion of *hyfBCDEF*	Amp
pTotal-*hyfG*^His^ Δ*hyfDEF*	As pTotal-*hyfG*^His^ with in-frame deletion of *hyfDEF*	Amp
pTotal-*hyfG*^His^ Δ*fdhF* Δ*hydN*	As pTotal-*hyfG*^His^ with deletion of *fdhF* and *hydN*	Amp
pTotal Δ*hyfG*	As pTotal-*hyfG*^His^ with in-frame deletion of *hyfG*	Amp
pSU-*hyfBC*	As pSU-PROM carrying *P. atrosepticum hyfBC* genes	Kan
pSU-*hyfB-F*	As pSU-PROM carrying *P. atrosepticum hyfBCDEF* genes	Kan
pSU-*hyfDEF*	As pSU-PROM carrying *P. atrosepticum hyfDEF* genes	Kan
pSU-*fdhF*	As pSU-PROM carrying *P. atrosepticum fdhF*	Kan
pSU-*fdhF*/*hydN*	As pSU-PROM carrying *P. atrosepticum fdhF* and *hydN*	Kan
pSU-*hyfG*	As pSU-PROM carrying *P. atrosepticum hyfG*	Kan
pSU-*hyfG-J*	As pSU-PROM carrying *P. atrosepticum hyfGHIJ genes*	Kan
pSU-*hycE*	As pSU-PROM carrying *E. coli hycE*	Kan

A two-step strategy was devised for cloning the gene cluster due to the presence of a unique BglII restriction site within the *hyfE* gene. First, the *hyfR-E* region was amplified by PCR and cloned into pT.7-Litmus (Amp^R^), which was followed by amplification of the *hyfE-fdhF* region by PCR and addition to the new vector. This resulted in the p-Total (Amp^R^) plasmid encoding the full *hyfR-fdhF* gene cluster (Fig. 1C; Table 2). The p-Total plasmid was sequenced and, most likely due to its construction by PCR, found to contain several point mutations that are listed in Supplementary Table S1. Note that no non-sense mutations were introduced. Next, the p-Total plasmid was modified by the addition of a 10-His tag sequence between codons for Gly-93 and Glu-94 within the *hyfG* gene primarily to facilitate future protein purification studies but potentially also to be used as an epitope tag in immunological studies. The location of the internal 10-His loop in HyfG was selected based on previous work conducted on *E. coli* FHL-1where identically-His-tagged HycE enabled the successful purification of the FHL-1 complex with minimal disruption to complex stability or activity [29]. Purification of FHL-2 is not an aim of this particular study, however characterisation of the in vivo physiological activity of His-tagged FHL-2 certainly is. Following construction, the pTotal-*hyfG*^His^ vector was sequenced and no additional mutations were identified.

To test if either the pTotal or pTotal-*hyfG*^His^ vectors encoded hydrogenase activity, FTD250 was transformed with the plasmids and anaerobic cultures were incubated in sealed Hungate tubes containing LB medium supplemented with 0.4% (w/v) glucose and 0.2% (w/v) formate. Following 20 h growth at 37 °C the headspace gas was analysed by gas chromatography (Fig. 2). FTD250[pTotal] produced 3.6 mM of hydrogen per OD, while FTD250[pTotal-*hyfG*^His^] produced 3.4 mM of H_2_ per OD (Fig. 2). These values were clearly lower than the 15.5 mM/OD of H_2_ produced in strain FTD89 that contains native FHL-1 (Fig. 2). Nevertheless, it was evident from these results that the cloned FHL-2 complex from *P. atrosepticum* was active in an *E. coli* host.

Next, it was pertinent to determine if FHL-2 could function in reverse as a hydrogen-dependent carbon dioxide reductase. To explore this, anaerobically-grown cultures were harvested and washed in MOPS buffer (pH 7.4) and sealed in Hungate tubes before being supplied with the gaseous substrates. Following incubation with a CO_2_/H_2_ mixture, clarified samples were analysed by HPLC to quantify formate levels in the aqueous phase. No formate was detected in experiments using FTD250[pTotal] or FTD250[pTotal-hyfG^His^] (Fig. 2). Conversely, the positive control experiment using FTD89 (producing active FHL-1) was capable of producing 4 mM formate under the same conditions (Fig. 2).

### The role of the membrane domain in FHL-2 function

**Fig. 3:**
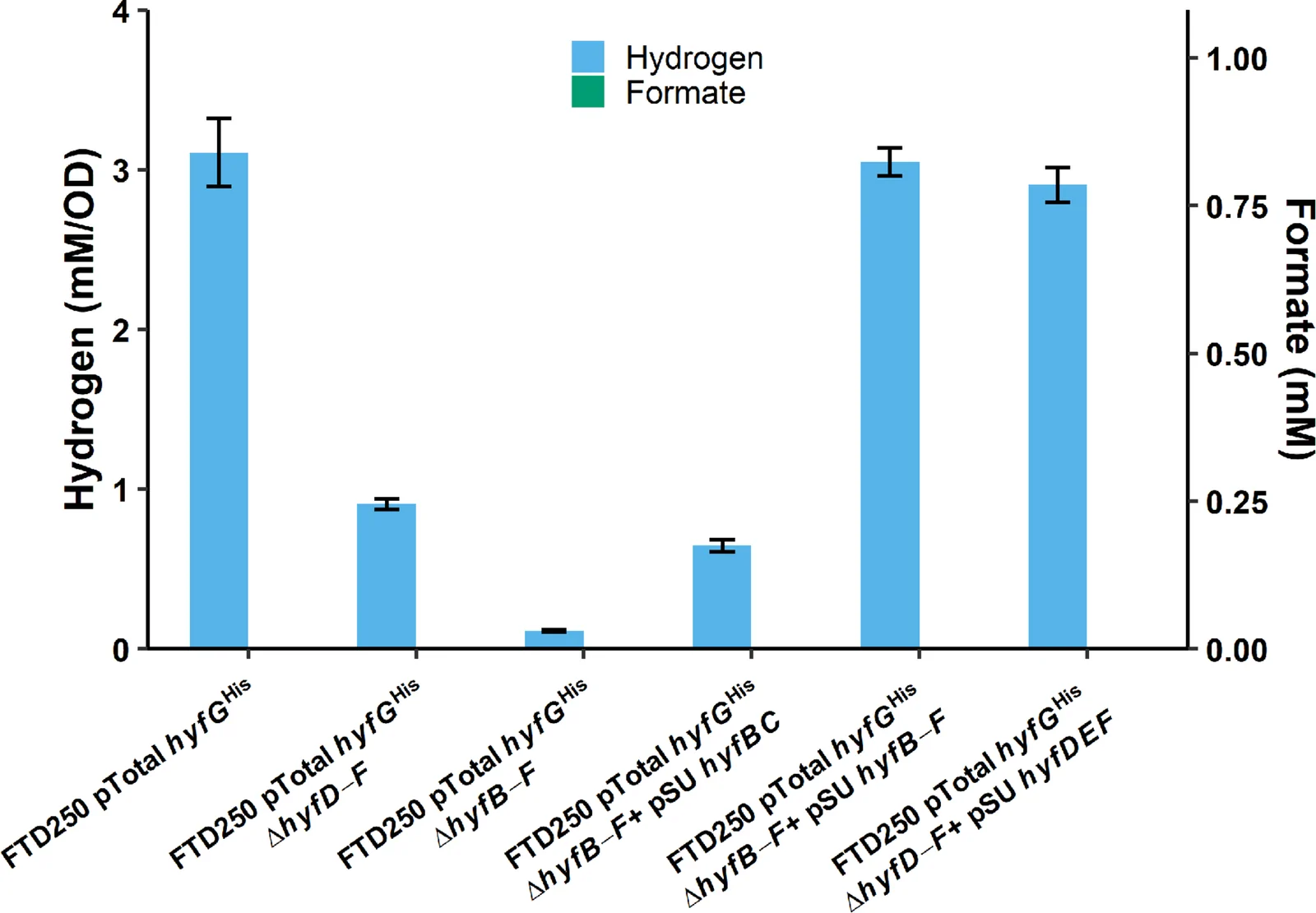
FHL-2 from *P. atrosepticum* requires a membrane arm for maximal activity. *E. coli* strain FTD250 was transformed with pTotal, expressing all the functional genes of FHL-2, or varients of the plasmid lacking genes encoding three membrane subunits (Δ*hyfD-F*) or all five membrane subunits (Δ*hyfB-F*), and cultured under anearobic fermentative conditions in LB medium supplimented with 0.4% (w/v) glucose and 0.2% (w/v) formate for 20 h at 37 °C (*n* = 3). Additionally, in trans complementation of the deleted genes was also tested using pSU-*hyfB-F* and pSU-*hyfDEF* plasmids. To determine hydrogen production activity (forward reaction) of the FHL complexes, cultures were grown in Hungate tubes to allow for hydrogen accumulation in the headspace. Hydrogen was removed from the headspace using a gas-tight, Luer-lock syringe and quantified by gas chromatography. To determine the formate production activity (reverse reaction) of the FHL complexes, anaerobic cultures were washed three times in MOPS buffer, pH 7.4 and resuspended to an OD 600 nm of 1.0 before being transferred to Hungate tubes and supplied with gaseous substrates. Cell suspensions were incubated at 37 °C for 20 h before sampleing. Formate production was quantified via HPLC. Error bars represent standard error of the mean

The FHL-2 complex is predicted to have major differences from FHL-1, including three additional membrane proteins (HyfDEF). To assess their role in recombinant FHL-2 activity mutant plasmids were constructed, including: pTotal *hyfG*^*His*^ Δ*hyfD-F* (lacking genes encoding HyfD, -E, and -F) and pTotal *hyfG*^*His*^ Δ*hyfB-F* (lacking genes encoding HyfB, -C, -D, -E, and -F) (Table 2). When the host strain FTD250 was transformed with pTotal *hyfG*^*His*^ *ΔhyfD-F* some FHL-2 activity was detected, however the mutant plasmid was found to produce less than 30% H_2_ than normally recorded for FTD250 expressing the whole FHL-2 complex (Fig. 3). The deletion of genes encoding all five membrane subunits of FHL-2 (Δ*hyfB-F*) resulted in an even greater reduction in hydrogen production activity, though it should be noted that trace levels of H_2_ could still be quantified here (Fig. 3).

Next, the genes encoding the FHL-2 membrane arm were cloned into the pSU-Prom vector (Kan^R^) (Table 2), which is compatible for co-expression with pLitmus (Amp^R^) derivatives. This enabled a series of *in trans* complementation experiments, which demonstrated full restoration of hydrogen production activity (Fig. 3).

Finally, hydrogen-dependent CO_2_-reduction experiments were carried out on FTD250 carrying pTotal *hyfG*^*His*^ Δ*hyfD-F* or pTotal *hyfG*^*His*^ Δ*hyfB-F*, but no formate was detected in any of the samples (Fig. 3).

### The role of formate dehydrogenase subunits in FHL-2 function

**Fig. 4:**
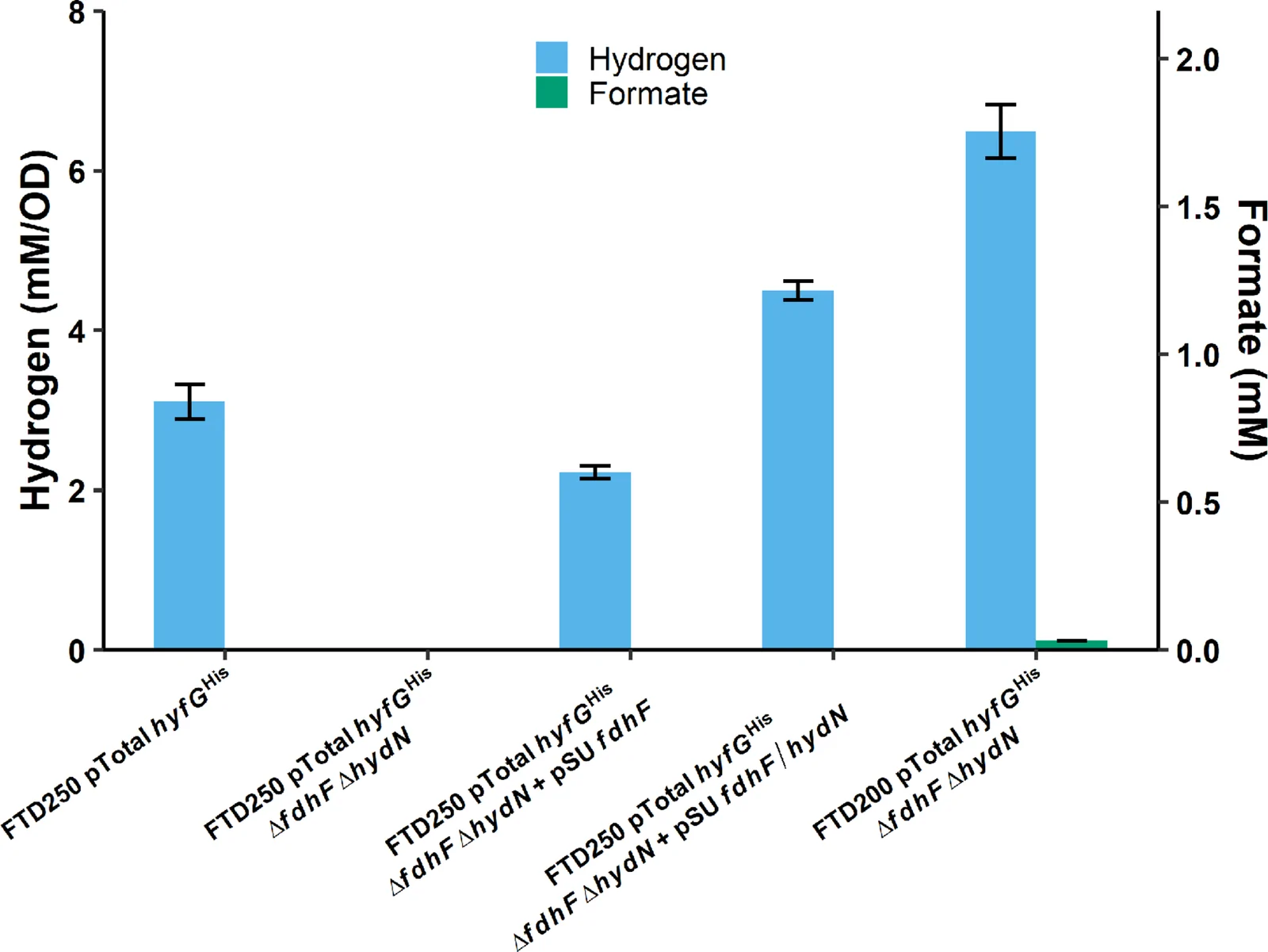
FdhF and HydN are both required for maximal activity of FHL-2 from *P. atrosepticum*. *E. coli* strain FTD250 was transformed with pTotal-*hyfG*^His^, expressing all the functional genes of FHL-2, or a varient of the plasmid lacking *fdhF* and *hydN*, was cultured under anearobic fermentative conditions in LB medium supplimented with 0.4% (w/v) glucose and 0.2% (w/v) formate for 20 h at 37 °C (*n* = 3). Additionally, in trans complementation of the deleted genes were also tested using pSU-*fdhF* and pSU-*fdhF*/*hydN*. The strain FTD200 transformed with pTotal *hyfG*^His^ Δ*fdhF* Δ*hydN* was also used in these experiments to determine if *E. coli* FdhF could function with Hyd-4 from FHL-2. To determine hydrogen production activity (forward reaction) of the FHL complexes, cultures were grown in Hungate tubes to allow for hydrogen accumulation in the headspace. Hydrogen was removed from the headspace using a gas-tight, Luer-lock syringe and quantified by gas chromatography. To determine the formate production activity (reverse reaction) of the FHL complexes, anaerobic cultures were washed three times in MOPS buffer, pH 7.4 and resuspended to an OD 600 nm of 1.0 before being transferred to Hungate tubes and supplied with gaseous substrates. Cell suspensions were incubated at 37 °C for 20 h before sampleing. Formate production was quantified via HPLC. Error bars represent standard error of the mean

Next, the importance of the *P. atrosepticum* formate dehydrogenase in FHL-2 activity was investigated. In *P. atrosepticum* the catalytic subunit of the formate dehydrogenase (*fdhF*) is encoded upstream of the *hyf* operon, on a divergent promoter, along with *hydN*. The HydN protein shares sequence similarity with HyfA and is therefore predicted to encode an alternative small subunit for the formate dehydrogenase. Therefore, a deletion in both *fdhF* and *hydN* together was made in the pTotal *hyfG*^*His*^ plasmid (Table 2). The resultant pTotal *hyfG*^*His*^ Δ*fdhF* Δ*hydN* vector was then tested in the FTD250 host strain and it was found that deletion of both *fdhF* and *hydN* together resulted in the complete loss of hydrogen production activity by FHL-2 (Fig. 4). Complementation *in trans* with pSU *fdhF* (Kan^R^), which carries only one gene, restored hydrogen production activity to ~ 71% of that of that normally recorded for the whole FHL-2 complex (Fig. 4). Furthermore, the full rescue of FHL-2 activity was observed when pTotal *hyfG*^*His*^ Δ*fdhF* Δ*hydN* was co-expressed with a second plasmid encoding both *fdhF* and *hydN* together (Fig. 4). No hydrogen-dependent CO_2_-reductase activity was detectible in any of these experiments (Fig. 4).

### Cross-complementation between FHL-1 and FHL-2 subunits: FdhF

*E. coli* strain FTD200 differs from FTD250 in that it contains a native *fdhF* gene. Both strains lack native hydrogenase activity and therefore produce no H_2_ *via* FHL-1, nor are they capable of producing formate via HDCR (Fig. 2). However, when FTD200 was transformed with pTotal *hyfG*^*His*^ Δ*fdhF* Δ*hydN* hydrogen production activity was rescued to a high level - almost double that recorded for intact FHL-2 in FTD250 (Fig. 4). This suggests *E. coli* FdhF from FHL-1 can interact with *P. atrosepticum* Hyd-4 from FHL-2. Moreover, it should be noted that trace but detectable amounts of formate (61 nM) were detected in samples of FTD200 pTotal *hyfG*^*His*^ Δ*fdhF* Δ*hydN* when incubated under an H_2_/CO_2_ atmosphere (Fig. 4). This represents only 1.5% of the total normally recorded in native FHL-1 experiments.

**Fig. 5:**
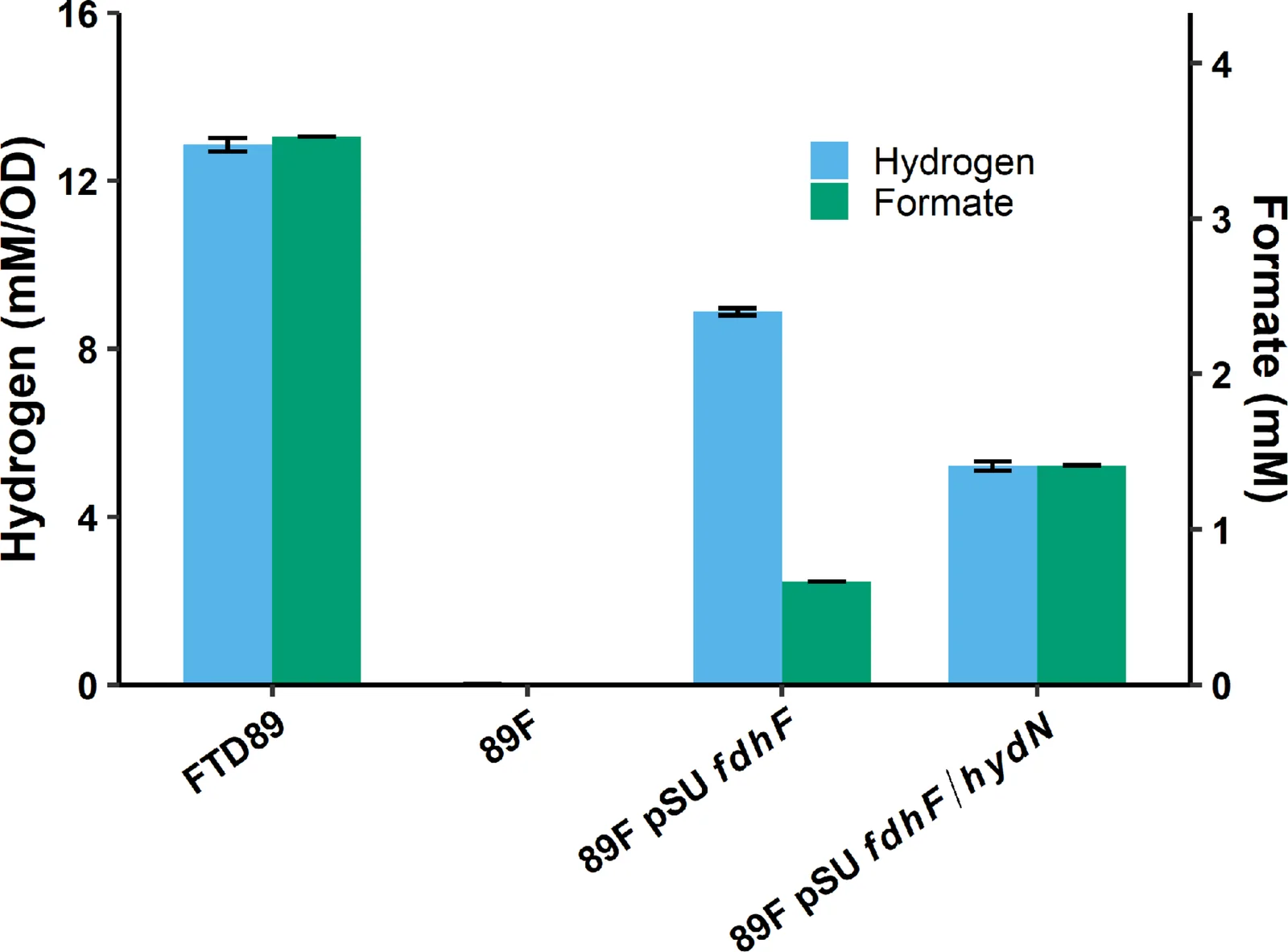
The formate dehydrogenase from *P. atrosepticum* FHL-2 can function as a Hydrogen-Dependent CO_2_ Reductase when in tandem with Hyd-3 from *E. coli*. *E.coli *strain FTD89 and 89F (as FTD89 Δ*fdhF*) were cultured under anearobic fermentative conditions in LB medium supplimented with 0.4% (w/v) glucose and 0.2% (w/v) formate for 20 h at 37 °C (*n* = 3). Additionally, in trans complementation was tested using the FHL-2 homolog with and without its predicted alternative small subunit, using plasmids pSU-*fdhF* and pSU-*fdhF*/*hydN*. To determine hydrogen production activity (forward reaction) of the FHL complexes, cultures were grown in Hungate tubes to allow for hydrogen accumulation in the headspace. Hydrogen was removed from the headspace using a gas-tight, Luer-lock syringe and quantified by gas chromatography. To determine the formate production activity (reverse reaction) of the FHL complexes, anaerobic cultures were washed 3 times in MOPS buffer, pH 7.4 and resuspended to an OD 600 nm of 1.0 before being transferred to Hungate tubes and supplied with gaseous substrates. Cell suspensions were incubated at 37 °C for 20 h before sampleing. Formate production was quantified via HPLC. Error bars represent standard error of the mean

Next, the ability of the formate dehydrogenase catalytic subunit from *P. atrosepticum* FHL-2 to substitute for that of native *E. coli* FHL-1 was tested. First, the *E. coli* strain 89F was constructed, which is devoid of Hyd-1 and Hyd-2 activity and carries a deletion in *fdhF* (Table 1). *E. coli*  89F displays no FHL-1-based H_2_ production activity (Fig. 5). When *E. coli* 89F was transformed with a plasmid encoding *P. atrosepticum* FdhF (pSU-*fdhF*) a partial restoration of hydrogen production activity was observed (Fig. 5). Interestingly, complementation with a plasmid containing both *P. atrosepticum fdhF* and *hydN* together resulted in less hydrogen production (5.2 mM/OD) than with the *fdhF* gene alone (8.8 mM/OD) (Fig. 5).

Hydrogen-dependent CO_2_ reduction experiments were also conducted in these strains (Fig. 5). As previously reported [8], *E. coli* strain FTD89, which has functional FHL-1, was able to generate formate from a H_2_/CO_2_ mixture at ambient pressure. Deletion of the gene encoding the formate dehydrogenase component of FHL-1 blocked this activity (Fig. 5). Complementation of strain 89F with *P. atrosepticum fdhF* alone, and in combination with *P. atrosepticum hydN*, produced chimeric complexes that were capable of HDCR activity and resulted in detectable formate generation from H_2_ and CO_2_ substrates (Fig. 5).

### Cross-complementation between FHL-1 and FHL-2 subunits: HyfG/HycE

**Fig. 6:**
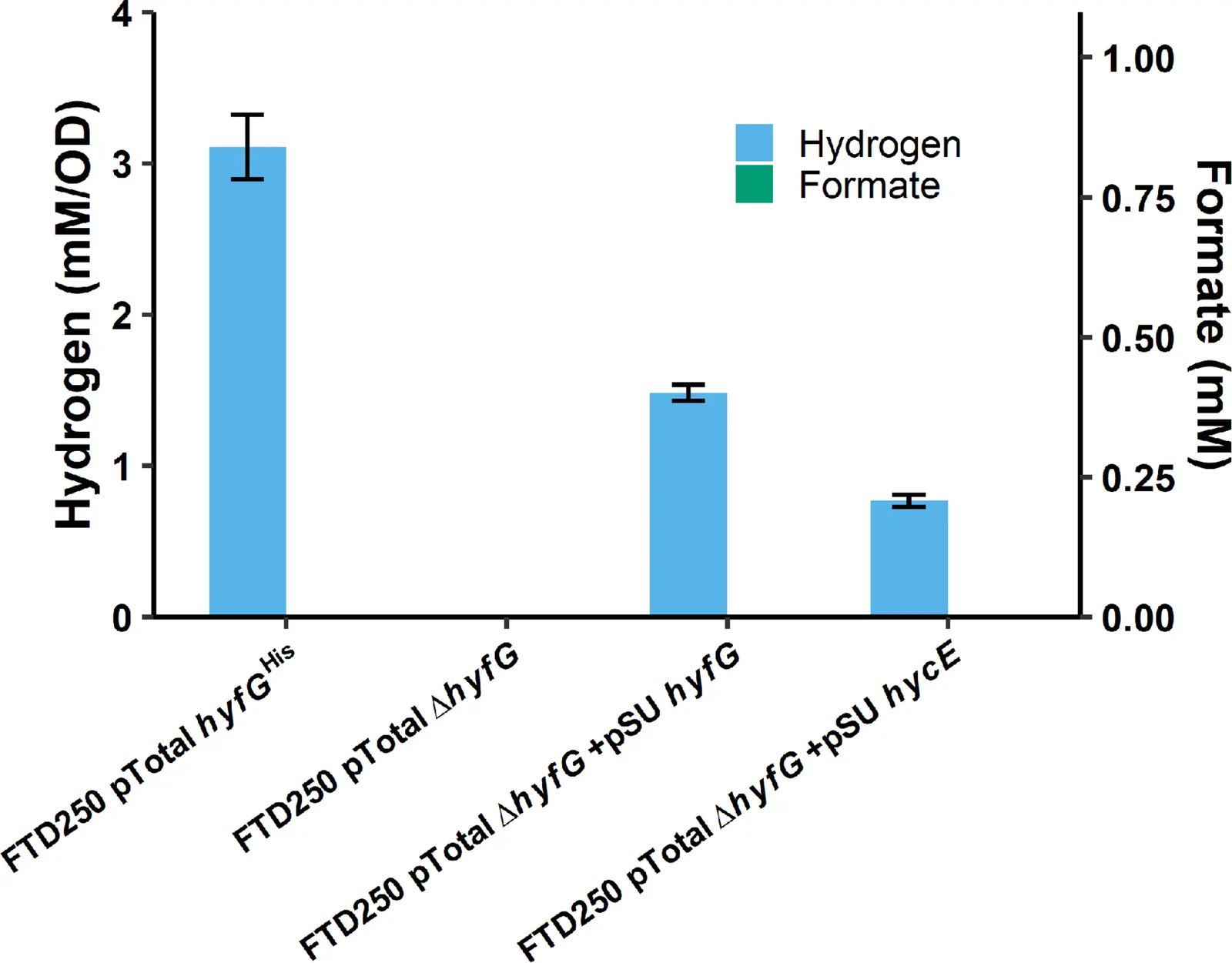
Tha large subunit of Hyd-4 is crutial for FHL-2 activity. *E. coli* strain FTD250 was transformed with pTotal-*hyfG*^His^, expressing all the functional genes of FHL-2, or a varient of the plasmid lacking the gene encoding the large subunit of hydrogenase-4 (*hyfG*) were cultured under anearobic fermentative conditions in LB medium supplimented with 0.4% (w/v) glucose and 0.2% (w/v) formate for 20 h at 37 °C (*n* = 3). Additionally, in trans complementation of the deleted gene was tested using pSU-*hyfG* and pSU-*hycE*, encoding the *E. coli* Hyd-3 large subunit. To determine hydrogen production activity (forward reaction) of the FHL complexes, cultures were grown in Hungate tubes to allow for hydrogen accumulation in the headspace. Hydrogen was removed from the headspace using a gas-tight, Luer-lock syringe and quantified by gas chromatography. To determine the formate production activity (reverse reaction) of the FHL complexes, anaerobic cultures were washed three times in MOPS buffer, pH 7.4 and resuspended to an OD 600 nm of 1.0 before being transferred to Hungate tubes and supplied with gaseous substrates. Cell suspensions were incubated at 37 °C for 20 h before sampleing. Formate production was quantified via HPLC. Error bars represent standard error of the mean

The possibility of cross-complementation between genes encoding the large subunits of Hyd-3 and Hyd-4 was investigated. A version of pTotal was prepared that carries an in-frame deletion of *hyfG* (pTotal Δ*hyfG*) (Table 2). The *E. coli* host strain FTD250 containing pTotal Δ*hyfG* was found to be devoid of hydrogen production activity (Fig. 6). A partial rescue in FHL-2 activity could be achieved through complementation *in trans* with a pSU-*hyfG* plasmid, and resulted in approximately half of the levels of H_2_ accumulation when compared to FHL-2 expressed from the intact operon. Interestingly, the gene encoding the large subunit of *E. coli* Hyd-3 (*hycE*) can also partially complement the *hyfG* deletion (Fig. 6). No hydrogen-dependent CO_2_-reductase activity was detectible in any of these experiments (Fig. 6).

**Fig. 7:**
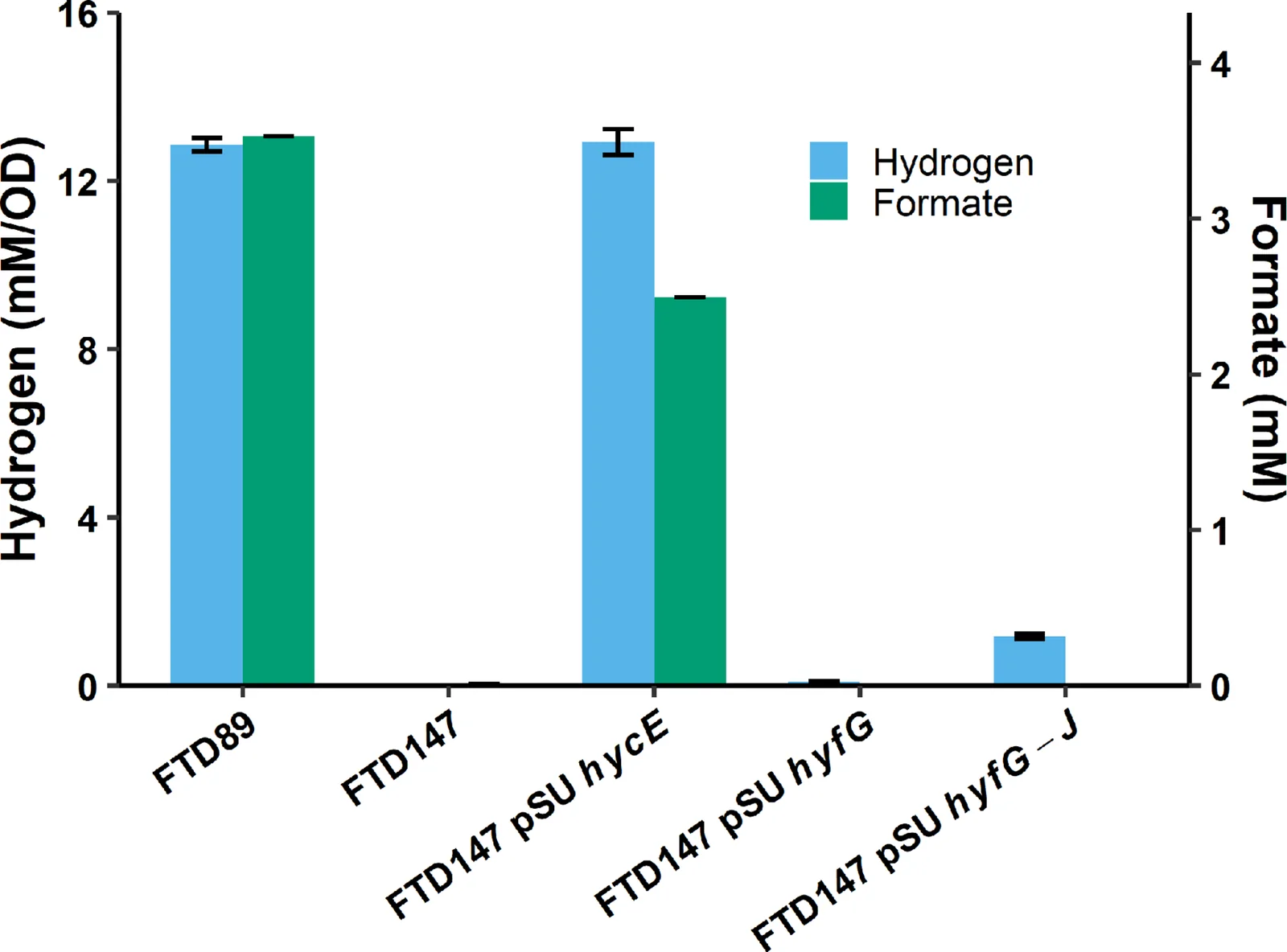
The Hyd-4 hydrogenase large subunit from FHL-2 cannot functionally interact with an FHL-1 variant lacking the Hyd-3 equivalent. *E. coli* Strain FTD89 and FTD147 (FTD89 Δ*hycE*) were cultured under anearobic fermentative conditions in LB medium supplimented with 0.4% (w/v) glucose and 0.2% (w/v) formate for 20 h at 37 °C (*n* = 3). Additionally, in trans complementation of the Δ*hycE* allele was tested using pSU-*hycE* (*E. coli* gene), and pSU-*hyfG* and pSU-*hyfGJ* (*P. atrospeticum* genes). To determine hydrogen production activity (forward reaction) of the FHL complexes, cultures were grown in Hungate tubes to allow for hydrogen accumulation in the headspace. Hydrogen was removed from the headspace using a gas-tight, Luer-lock syringe and quantified by gas chromatography. To determine the formate production activity (reverse reaction) of the FHL complexes, anaerobic cultures were washed 3 times in MOPS buffer, pH 7.4 and resuspended to an OD 600 nm of 1.0 before being transferred to Hungate tubes and supplied with gaseous substrates. Cell suspensions were incubated at 37 °C for 20 h before sampleing. Formate production was quantified via HPLC. Error bars represent standard error of the mean

Next, it was determined whether the hydrogenase large subunit of *P. atrosepticum* FHL-2 (HyfG) was capable of interacting with *E. coli* FHL-1. To investigate this, the *E. coli* strain FTD147, which carries a Δ*hycE* allele and is devoid of H_2_ production activity (Fig. 7), was transformed with pSU-*hyfG* encoding *P. atrosepticum* HyfG. Only a trace amount of molecular hydrogen gas was detected in fermentative cultures of FTD147[pSU *hyfG*] (Fig. 7). A plasmid was also tested that not only encoded *P. atrosepticum* HyfG but also encoded the FHL-2 small hydrogenase subunit (HyfH), an iron sulfur cluster protein related to HycF (HyfI), and a predicted Hyd-4 maturase related to HycH (HyfJ). This pSU *hyfG-J* vector produced small (~ 9% of the positive control) but detectible levels of H_2_ when expressed in the *E. coli* FTD147 host (Fig. 7). No hydrogen-dependent CO_2_-reductase activity was detectible in any of the plasmid-based experiments (Fig. 7).

### Cross complementation between FHL-1 and FHL-2 subunits: membrane domain

**Fig. 8:**
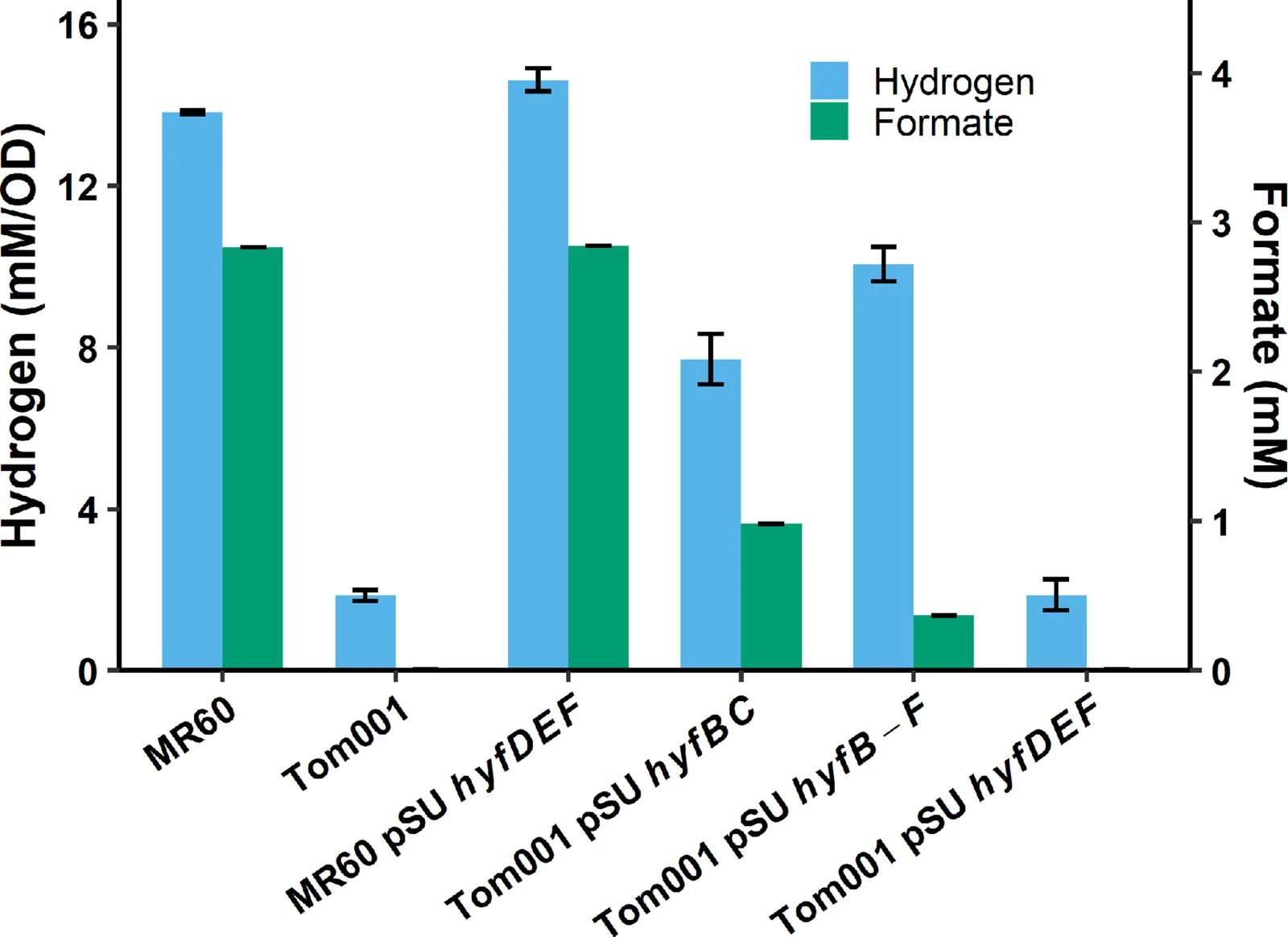
The membrane subunits of FHL-2 can interact with the peripheral domain of FHL-1. E. coli strain MR60 and Tom001 (MR60 ΔhycCD) were transformed with plasmids encoding various combinations of the membrane subunits of P. atrosepticum FHL-2, including pSU-hyfBC, pSU-hyfDEF and pSU-hyfB-F (encoding all five subunits). Strains were cultured under anearobic fermentative conditions in LB medium supplimented with 0.4% (w/v) glucose and 0.2% (w/v) formate for 20 h at 37 °C (*n* = 3). To determine hydrogen production activity (forward reaction) of the FHL complexes, cultures were grown in Hungate tubes to allow for hydrogen accumulation in the headspace. Hydrogen was removed from the headspace using a gas-tight, Luer-lock syringe and quantified by gas chromatography. To determine the formate production activity (reverse reaction) of the FHL complexes, anaerobic cultures were washed three times in MOPS buffer, pH 7.4 and resuspended to an OD 600 nm of 1.0 before being transferred to Hungate tubes and supplied with gaseous substrates. Cell suspensions were incubated at 37 °C for 20 h before sampleing. Formate production was quantified via HPLC. Error bars represent standard

A key point of similarity, and a key point of difference, between FHL-1 and FHL-2 is the membrane domain. FHL-1 contains two subunits in the membrane domain, HycCD. The equivalent proteins exist in FHL-2 (HyfBC), with HyfD, -E, and -F being additional components. First, it was determined if the membrane domain of *P. atrospeticum* FHL-2 could complement the *E. coli* FHL-1 complex lacking its own membrane domain. *E. coli* strains MR60 (contains active FHL-1) and Tom001 (as MR60 but Δ*hycCD*) were transformed with constructs containing FHL-2 membrane subunits in varying combinations before hydrogen and formate production activities were determined (Fig. 8). The control experiments with MR60 and Tom001 alone displayed activities consistent with previous research [30], where the Δ*hycCD* retains a low level of H_2_ accumulation activity compared to the parent strain. When *E. coli* Tom001 was transformed with pSU-*hyfBC* a partial restoration of hydrogen production activity was observed reaching ~ 53% of that normally recorded for native FHL-1 (Fig. 8). Transformation of Tom001 with pSU-*hyfDEF*, encoding the three membrane subunits of FHL-2 not found in FHL-1, could not rescue H_2_ production activity (Fig. 8). Taken together, this showed a functional chimera could be formed using the soluble arm of FHL-1 and core conserved subunits from the membrane arm of FHL-2.

Next, *E. coli* Tom001 was transformed with pSU-*hyfB-F* encoding all five subunits of the *P. atrosepticum* FHL-2 membrane arm. In this case hydrogen production activity was observed to a level of ~ 76% that normally recorded for FHL-1 (Fig. 8).

The hydrogen-dependent CO_2_-reduction ‘reverse’ activities were also explored in these strains. Interestingly, Tom001 carrying either pSU-*hyfBC* or pSU-*hyfB-*F was found to be capable of formate production from H_2_/CO_2_ (Fig. 8). In both cases the formate production activity was less than 35% of that of the native FHL-1 complex from the positive control strain MR60. However, comparing the two, formate production activity was significantly greater for the strain carrying pSU-*hyfBC* compared to that carrying the larger construct pSU-*hyfB-F* (Fig. 8).

## Discussion

### *P. atrosepticum* FHL-2 is functional in an *E. coli* host

In this work, we set out to identify a suitable, physiologically active FHL-2 that could be produced in *E. coli* to allow direct comparison with the native FHL-1 isoenzyme. Here, it has now been shown that the *P. atrosepticum* FHL-2 is functional in an *E. coli* host. This was enabled by constructing an *E. coli* host strain (FTD250) devoid of the majority of structural genes involved in hydrogen metabolism, including the native *hycABCDEFGHI* and *hyfABCDEFGHIJ-hyfR* operons and the *fdhF* gene, known or predicted to be involved in the formate hydrogenlyase reaction. This allowed unambiguous characterisation of a cloned, recombinant version of *P. atrosepticum* FHL-2. The work with pTotal and its derivatives (Table 2) has clearly established that the enzyme can be produced and assembled at 37 °C and that the biosynthetic machineries for *E. coli* [NiFe] hydrogenases and *E. coli* molybdo-enzymes can facilitate maturation of the enzymes (Fig. 2).

### *P. atrosepticum* formate dehydrogenase (FdhF) is bidirectional

In this work, the FHL-2 complex from *P. atrosepticum* was clearly shown to be able to contribute to H_2_ production under anaerobic fermentative conditions (the ‘forward’ reaction). However, unlike *E. coli* FHL-1, *P. atrosepticum* FHL-2 was unable to function in reverse as a hydrogen-dependent CO_2_ reductase, at least under the conditions tested here (Fig. 2). A series of genetic experiments were employed to begin to dissect the molecular basis of this unidirectionality.

The role of the *P. atrosepticum* formate dehydrogenase component was investigated. Indeed, sequence analysis of this protein, and genomic analysis of the organism, suggest it does not contain selenocysteine at its active site. This is interesting since substitution of the selenocysteine for a cysteine residue within the *E. coli* FdhF active site has been shown to significantly reduce its in vitro CO_2_ reduction activity to 19% of the native enzyme [34]. In our experiments, the selenium-free *P. atrosepticum* FdhF was shown to be readily reversible in vivo, but only when paired with the *E. coli* Hyd-3 enzyme (Fig. 5). The isolated *P. atrosepticum* FdhF was able to substitute for the *E. coli* enzyme in FHL-1 in both the H_2_ production and formate production directions, meaning the absence of selenocysteine is not regulating directionality of this component of FHL-2.

The interchangeability of the different species’ FdhF subunits was perhaps not entirely surprising. The *P. atrosepticum* FdhF shares 85% overall sequence similarity with the *E. coli* FHL-1 counterpart (Supplementary Figures S1-S10). Moreover, the interaction between *E. coli* FdhF and the remainder of the *E. coli* FHL-1 complex is known to be transient and weak and the proteins readily dissociated upon purification, especially under reducing conditions [32]. Structural studies have determined that *E. coli* FdhF interacts with *E. coli* HycB though hydrogen bonding and hydrophobic interactions. Additionally, a single electrostatic interaction with Arg-122 of *E. coli* HycF was also noted. The data presented in this work would suggest that at least some of these interactions are conserved between FHL-1 and FHL-2. Lindenstrauß & Pinske (2019) have also demonstrated that the *E. coli* FdhF can form a functional complex with the Hyd-4 complex of *T. guamensis*, where the chimeric complex demonstrated the same level of hydrogen production as the native FHL-2 complex [23].

### HydN is a functional subunit of the FHL-2 complex

The HydN protein is a small Fe-S protein with sequence identity to HyfA of FHL-2 and HycB of FHL-1 (Supplementary Figures S1-S10). Its possible role as a formate dehydrogenase small subunit has long been speculated upon, however the genetics and biochemistry of *E. coli* added confusion. In *E. coli*, *hydN* is encoded with a hydrogenase maturation gene *hypF* [35]. Deletion of the *hydN* gene in *E. coli* led to an slight reduction in dye-linked formate dehydrogenase activity [35] but overall FHL-1 activity was not significantly affected [36]. Indeed, *E. coli* FdhF can be purified in an active form without a small subunit [11] and HydN was not present in the *E. coli* FHL-1 cryo-EM structure [12]. The data presented in Figs. 4 and 5 show the functional importance of HydN and these data suggest that the cognate HydN studied here, along with FdhF, might have some influence in the directionality of the FHL-2 complex. Extra experimentation would be needed to explore this further, however, since the *E. coli hydN* gene is present in all of these experiments.

### *P. atrosepticum* Hyd-4 is unidirectional

The unidirectionality of FHL-2 seems to stem for the most part from the Hyd-4 module, and most specifically the membrane arm. Deletion of the genes encoding the membrane arm from the native FHL-2 resulted in a loss in H_2_ evolution activity (Fig. 3). This is consistent with the behaviour of FHL-1 when its membrane arm is genetically removed [13]. More interestingly, it is shown here that the FHL-2 membrane arm, either in minimal HyfBC form (which is the equivalent to HycCD in FHL-1) or in extended HyfBCDEF form, can interact with the FHL-1 peripheral arm and restore H_2_ evolution activity (Fig. 8). However, it is clear that the reverse formate production activity is not restored in the same FHL-1/FHL-2 chimeras (Fig. 8). There, the presence of the full-length HyfBCDEF membrane arm reduces the ability of the enzyme to operate in reverse quite significantly (Fig. 8).

It should also be considered that changes in oxygen sensitivity could also contribute to these observations. No strict anaerobic precautions were put in place during the cell washing stage prior to anaerobic incubation with CO_2_/H_2_. While native FHL-1 survives this treatment, it could be that FHL-2, and/or the FHL-1/FHL-2 chimeras, are more sensitive to O_2_ attack.

The molecular basis of the interaction between the FHL-1 membrane arm and peripheral arm is now well understood. Structural data obtained for *E. coli* FHL-1 revealed the presence of a cluster of loops at the interface that are conserved across the complex I and complex I-like families, including membrane-bound hydrogenases [12]. Indeed, previous research has shown the disruption of glutamate residues within the loop that connects HycE with HycD can have significant negative effects on FHL activity [32, 37]. It should be considered that, in our chimeric FHL complexes, incorrect or unstable binding of peripheral arm to the membrane arm may be the basis of the unidirectionality observed. Indeed, further analysis into the native structure of FHL-2 will be required to effectively compare the loop-cluster structure and wider interaction interface.

Experiments focused on the hydrogenase catalytic subunits did not produce meaningful data (Figs. 6 and 7). This may not be altogether surprising when it is considered that these subunits are normally buried in the middle of a much larger complex with at least four other interacting partners to consider. Even cross-substitution of relatively conserved hydrogenase large and small subunits may not be straightforward. For example, a similar occurrence has been noted when attempting to form chimera of *E. coli* hydrogenase-1 and hydrogenase-2 [38]. To facilitate the assembly of chimeras between these two isoenzymes, a total of 13 residues required engineering at the large subunit – small subunit interface [39].

Many hydrogenases function bidirectionally and are capable of catalysing reactions reversibly [32, 39, 40]. Furthermore, Mo/W-dependent formate dehydrogenases are known to catalyse the oxidation of formate to CO_2_ reversibly [41]. However, bidirectionality is not uniform across these enzyme families. O_2_-tolerant [NiFe]-hydrogenases, such as *E. coli* Hyd-1, are generally thought to function unidirectionally in H_2_-uptake [42]. Several other [NiFe]-dependent hydrogenases have shown catalytic bias for hydrogen oxidation with little to no proton reduction activity [43–45]. Interestingly, *Pyrococcus furiosus* possesses a [NiFe] dependent membrane-bound hydrogenases that preferentially catalyses H_2_ production and displays very little H_2_ oxidation activity [46]. Outwith the O_2_-tolerance mechanism observed in some [NiFe] hydrogenases, and the properties of the metal centres and gas channels that are involved, the reversibility of metalloenzymes either in vitro or in vivo is difficult to predict.

## Concluding remarks

In this work, the FHL-2 complex from *P. atrosepticum* was cloned and shown to be active in an *E. coli* host and capable of producing molecular hydrogen during fermentative growth. Furthermore, we have shown that this FHL-2 complex appears to function unidirectionally in favour of hydrogen production. The inability of FHL-2 to function in reverse appears to stem from subtle molecular differences in the Hyd-4 module and the presence of additional subunits in the membrane arm. There may also be some involvement with the HydN-containing formate dehydrogenase module. Indeed, this work identified HydN as an bona fide alternative formate dehydrogenase small subunit in FHL-2 and future work will focus on understanding the competing or complementary role of HydN with its homolog HyfA within the complex. Indeed, the molecular tools developed here will allow further characterisation of important aspects of FHL-2 activity, including the role the complex I-like membrane arm. Understanding how electron transport is coupled to proton translocation in membrane-bound hydrogenases has wider significance since complex-I-like enzymes are found in a broad range of microbes and eukaryotic organelles.

## Supplementary Information

Below is the link to the electronic supplementary material.

Supplementary Material 1

Supplementary Material 2

## Data Availability

No datasets were generated or analysed during the current study.
