# Supplementary material for: Genetic dissection of formate hydrogenlyase-2 function: a metalloenzyme involved in bacterial hydrogen production
Source: J Biol Inorg Chem. 2026 Jul 23;31(6):521–35. doi: 10.1007/s00775-026-02168-7 (PMC13645896; doi:10.1007/s00775-026-02168-7)
Supplement: Supplementary file 1 [file 775_2026_2168_MOESM1_ESM.docx]

**SUPPLEMENTARY TABLE S1**

Genetic dissection of formate hydrogenlyase-2 function: a metalloenzyme involved in hydrogen production.

Thomas C.P. Reed, Alexander J. Finney, Magali Roger, Frank Sargent

| **Plasmid name** | **Gene** | **Codon change*** |
| --- | --- | --- |
| pTotal | *hyfA* | L49L (silent) |
|  | *hyfB* | M24T  R76C  K371R  L425P |
|  | *hyfD* | P377S |
|  | *hyfF* | W52R  M138T  L487P  S534G |
|  | *hyfI* | S74P |
|  | *hydN* | V147A |
| pTotal-*hyfG*^His^ | no changes from pTotal | no changes from pTotal |
|  |  |  |

*sequence changes as compared with genome of *Erwinia carotovora* subsp. *atroseptica* SCRI1043
