# Supplementary material for: Genetic dissection of formate hydrogenlyase-2 function: a metalloenzyme involved in bacterial hydrogen production
Source: J Biol Inorg Chem. 2026 Jul 23;31(6):521–35. doi: 10.1007/s00775-026-02168-7 (PMC13645896; doi:10.1007/s00775-026-02168-7)
Supplement: Supplementary file 2 [file 775_2026_2168_MOESM2_ESM.docx]

**SUPPLEMENTARY FIGURES**

Genetic dissection of formate hydrogenlyase-2 function: a metalloenzyme involved in bacterial hydrogen production.

Thomas C.P. Reed, Alexander J. Finney, Magali Roger, Frank Sargent

```
P._atrosepticum_FdhF      MKKITSVCPYCGAGCKLKLVVEDNKIIRAEAADGVTNQNELCLKGYYGWDFLNDTQLLTPRLTQPMIRYEKGGRLTPVSWDEAIRYTAQRFSEIKAKYGPRSIMTTGSSRGTGNETNYVM	120
E._coli_FdhF              MKKVVTVCPYCASGCKINLVVDNGKIVRAEAAQGKTNQGTLCLKGYYGWDFINDTQILTPRLKTPMIRRQRGGKLEPVSWDEALNYVAERLSAIKEKYGPDAIQTTGSSRGTGNETNYVM	120
                          ***:.:*****.:***::***::.**:*****:* ***. ***********:****:*****. **** ::**:* *******:.*.*:*:* ** **** :* ****************

P._atrosepticum_FdhF      QKFARAVLNTNNVDCCARVCHGPSVAGLQEVLGNGAMSNSISDIENSKCLLIFGYNSADSHPIVARRVVKAHQNGAKIIVCDPRRIEAARIADQHLQLNNGSNMALVNAFGYVLLEEKLY	240
E._coli_FdhF              QKFARAVIGTNNVDCCARVUHGPSVAGLHQSVGNGAMSNAINEIDNTDLVFVFGYNPADSHPIVANHVINAKRNGAKIIVCDPRKIETARIADMHIALKNGSNIALLNAMGHVIIEENLY	240
                          *******:.********** ********:: :*******:*.:*:*:. :::**** ********.:*::*::***********:**:***** *: *:****:**:**:*:*::**:**

P._atrosepticum_FdhF      NQHYVDRFTEGLDTYREIVKDYAPEAVEHLTGVPAADVRQAMRTFAAAPSATIMWGMGVTQFGQAVDVVRGLAGLALLTGNLGRENVGVGPIRGQNNVQGACDMGVLPNMFPGYQSVTDP	360
E._coli_FdhF              DKAFVASRTEGFEEYRKIVEGYTPESVEDITGVSASEIRQAARMYAQAKSAAILWGMGVTQFYQGVETVRSLTSLAMLTGNLGKPHAGVNPVRGQNNVQGACDMGALPDTYPGYQYVKDP	360
                          :: :*   ***:: **:**:.*:**:**.:*** *:::*** * :* * **:*:******** *.*:.**.*:.**:******: :.**.*:*************.**: :**** *.**

P._atrosepticum_FdhF      DVRAKFADAWGISPAVMDENIGTRITEVPHMALEGKIKAYYIMGEDPLQTEADLSLVRRGVEALDFLVVQDIFMTKTAEVADVLLPATSWGEHGGVFTCADRGFQRFEKAIEPKGNVKRD	480
E._coli_FdhF              ANREKFAKAWGVESL--PAHTGYRISELPHRAAHGEVRAAYIMGEDPLQTDAELSAVRKAFEDLELVIVQDIFMTKTASAADVILPSTSWGEHEGVFTAADRGFQRFFKAVEPKWDLKTD	478
                            * ***.***:.      : * **:*:** * .*:::* **********:*:** **:..* *::::**********..***:**:****** ****.******** **:*** ::* *

P._atrosepticum_FdhF      WEIISLIASEMGYPMHYADNQQIWDEMRSLCSLFYGVTYEKMGDMGHIQWPCPTLDH--PGTPYLYQDSHFDTPTGKGKLFATQWRAPAEVPDEAYPLVLCTVREVGHYSCRSMTGNCAA	598
E._coli_FdhF              WQIISEIATRMGYPMHYNNTQEIWDELRHLCPDFYGATYEKMGELGFIQWPCRDTSDADQGTSYLFK-EKFDTPNGLAQFFTCDWVAPIDKLTDEYPMVLSTVREVGHYSCRSMTGNCAA	597
                          *:*** **:.******* :.*:****:* **  ***.******::*.*****   ..   ** **:: .:****.* .::*: :* ** :   : **:**.*******************

P._atrosepticum_FdhF      LQALADEPGRVQMHPQDANRLGIKDRSLVWVSSRRGKVISRADISERINKGTIYMTYQWWIGACNELTQDNLDPISKTPETKYCAAKVDVIEDQRWAEDFASTSYNDMKARLRAAVAH	716
E._coli_FdhF              LAALADEPGYAQINTEDAKRLGIEDEALVWVHSRKGKIITRAQVSDRPNKGAIYMTYQWWIGACNELVTENLSPITKTPEYKYCAVRVEPIADQRAAEQYVIDEYNKLKTRLREAALA	715
                          * ******* .*:: :**:****:*.:**** **:**:*:**::*:* ***:***************. :**.**:**** ****.:*: * *** **::.  .**.:*:*** *.
```

**Supplementary Figure S1: Sequence alignment of formate dehydrogenase subunits.**

The relative location of *E. coli* FdhF selenocysteine residue at position 140 (U) is highlighted in yellow. The *P. atrosepticum* FdhF does not have this, but instead standard cysteine is located here.

```
P._atrosepticum_HydN      MMNRFVIVDSSKCIGCRTCEVACVVSHQEAQDCASLTQENFLPRIHVIKGANVSTAVMCRQCEDAPCANVCPNGAITREKDFIHVQQARCIGCKTCVVACPYGVMEVVSR-PVM------	113
E._coli_HycB              -MNRFVIADSTLCIGCHTCEAACSETHRQH-------GLQSMPRLRVMLNEKESAPQLCHHCEDAPCAVVCPVNAITRVDGAVQLNESLCVSCKLCGIACPFGAIEFSGSRPLDIPANAN	112
P._atrosepticum_HyfA      -MNRFVIADPELCIGCNTCMAGCTSVHKAQ-------GLQGLPRLTVVKTEDKTAPLMCRQCEDAPCARVCPVNAITHENAAIVLNESLCIGCKLCGLVCPFGAITPSGSTPVNTPALLQ	112
                           ******.*   ****.** ..*   *:           : :**: *:   . ::  :*::******* *** .***: .  : :::: *:.** * :.**:*.:   .  *:

P._atrosepticum_HydN      ------------KKRITGEPFLSY----KVEANKCDLCDGHAEGPACINVCPTNAIVSIDRDKLEYMNVEKRRRAALNSIEILNF----------	182
E._coli_HycB              TPK----APPAPPAPARVSTLLDWVPGIRAIAVKCDLCSFDEQGPACVRMCPTKALHLVDNTDIARVSKRKRELTFNTDFGDLTLFQQAQSGEAK	203
P._atrosepticum_HyfA      NMIPEALLRDVPGSAPGTNPFIAWNAGIRAIAVKCDLCDFQDTGPECIRVCPTKSISLVDSQSISTNSQSRRLKTLLSSFHEQGFMSVQQEGDE-	206
                                            . :: :    :. * *****. .  ** *:.:***:::  :*  .:   .  :*  :  ..:    :
```

**Supplementary Figure S2: Sequence comparison of formate dehydrogenase small subunits.**

The *P. atrosepticum* HyfA Fe-S protein is encoded within the Hyd-4 operon, while *P. atrosepticum* *hydN* is expressed upstream of the formate dehydrogenase catalytic subunit *fdhF*. *E. coli* HycB is known to be part of FHL-1 via structural studies.

```
E._coli_NuoL              MNM---LALTIILPLIGFVL-LAFSR----GRWSENVSAIVGVGSVGLAALVTAFIGVDFFANGEQTYSQPLWTWMSVGDFN-IGFNLVLDGLSLTMLSV----VTGVGFLIHMYA-SWY	106
P._atrosepticum_HyfB      MDSLQLLQWSAICYLAGAVLSLLMARKENASIIIAAITAICGG-------VLGLCSAVPVLLSGQI------QLYATQGLFHFATFTVRLDMLAAFMVTVISLLVTVCSLYSIAYVREYI	107
E._coli_HycC              MSAISLINSGVAWFVAAAVLAFLFSFQKALSGWIAGIGGAVGS-------LYTAAAGF-TVLTGAV---------GVSGALSLVSYDVQISPLNAIWLIT----LGLCGLFVSLYNIDWH	99
                          *.    :       : . ** : ::     .     : .  *        :     .. . .*               * :    : : :. *    : .    :   .:    *  .:

E._coli_NuoL              MRGEEGYSRFFAYTNLFIASMVVLVLADNLLLMYLGWEGVGLCSYLLIGFYYTDPKNGAAAMKAFVVTRVGDVFLAFALFILYNELGTLNFREMVELAPAHFADGNNMLMWATLMLLGGA	226
P._atrosepticum_HyfB      GRGAGS--M-GGFMNLFIASMVGLVVMDNAFYFIILFEAMSLASWFLVISE-QDRESVSAGLLYFFIAHAGSVLIMIAFFLMWRESGSLDFAAFRRLSLSPVLSSV---VFL--LAFFGF	218
E._coli_HycC              RHAQVK--CNGLQINMLMAAAVCAVIASNLGMFVVMAEIMALCAVFLTS-------NSKEGKLWFALGRLGTLLLAIACWLLWQRYGTLDLRLLDMRMQQLPLGSD---IWL--LGVIGF	205
                           :.           *:::*: *  *: .*   : :  * :.*.: :*         .   .   * : : * ::: :* ::::.. *:*::  :         ..    ::   : . *

E._coli_NuoL              VGKSAQLPLQTWLADAMAGPTPVSALIHAATMVTAGVYLIARTHGLFLMTPEVLH--LVGIVGAVTLLLAGFAALVQTDIKRVLAYSTMSQIGYMFLALGVQAW--------------DA	330
P._atrosepticum_HyfB      GAKAGMLPLHSWLPRAHPAAPSHASALMSGVMVKIGIFGIIKVGIDLLAAPQLWWGIVVLAFGSISAVLGVLYALAEHDIKRLLAWHTVENVGIILMGVGVGMVGIATQHQVLATLGLLG	338
E._coli_HycC              GLLAGIIPLHGWVPQAHANASAPAAALFSTVVMKIGLLGILTLSLL-GGNAPLWWGIALLVLGMITAFVGGLYALVEHNIQRLLAYHTLENIGIILLGLGAGVTGIALEQPALIALGLVG	324
                             :. :**: *:  *       :: : : .::. *:  *            :      : .* :: .:. : **.: :*:*:**: *:.::* :::.:*.                  .

E._coli_NuoL              AIFHLMTHAFFKALLFLASGSVILACHHEQNIFKMGGLRKSIPLVYLCFLVGGAALSALPLVTAGFFSKDEILAGAMANGHINLMVAGLV-----------GAFMTSLYTFRMIFIVFHG	439
P._atrosepticum_HyfB      ALYHLLNHAVFKGLLFLGAGAVIYRLHT-RDMEKMGGLGKLMPKTALAFLIGCMSISALPPLN-GFISEWYTYQSLFSMSHDGNVLMRLSAPVAIVMLAITGALAA-MCFVKVYGVSFCG	455
E._coli_HycC              GLYHLLNHSLFKSVLFLGAGSVWFRTGH-RDIEKLGGIGKKMPVISIAMLVGLMAMAALPPLN-GFAGEWVIYQSFFKLSNSGAFVARLLGPLLAVGLAITGALAV-MCMAKVYGVTFLG	441
                          .::**:.*:.**.:***.:*:*       ::: *:**: * :*   :.:*:*  :::*** :. ** .:     . :  .: . .:  *            **: . :   ::  : * *

E._coli_NuoL              KEQIHA-HAVKGVT--HSLPLIVLLILSTFVGALIVPPLQ--------------------GVLPQTTELAHGSMLTLEITSGVVAVVGILLAAWLWLGKRTLVTSIANSAPGRLLGTWWY	536
P._atrosepticum_HyfB      APRSEEASQAREVPWQMTLSLLL-LALCCVALGLGASKVAPIIAQVAMSLSGAQDVAVAQGNLLIPAQAEQAMFSPALVFILLIALPVLPLLIWLFL--------KGNRPAFRRHGDPWA	566
E._coli_HycC              APRTKEAENATCAPLLMSVSVVA-LAICCVIGGVAAPWLLPMLSAA----------------VPLPLEPANTTVSQPMITLLLIACPLLPFIIMAIC--------KGDRLPSRSRGAAWV	536

                            : .    .  .    :: ::  * :. .  .: .  :                    :    :  :  .    :   ::*   : :               .:    *  *  *

E._coli_NuoL              NAWGFDWLYDKVFVKPFLGIAWLL---------KRDPLNSMMNIPAVLSRFA----------------------------GKGLLLSENGYLRWYVASMSIGAVVVLALLMVLR	613
P._atrosepticum_HyfB      C--GYAWEPQMV--VSASGFTQPLRAMFGGLYRMRQRLNPAPQLSRALQGITQGATIIEPFWDEKIIYP---LVRLVRRIGAQLQRLQGGDFRLYCLYVVAALTVLLLIVAV--	671
E._coli_HycC              C--GYDHEKSMV--ITAHGFAMPVKQAFAPVLKLRKWLNPVSLVPGWQC---EGSALL---FRRMALVELAVLVVIIVSRGA--------------------------------	608
                             *:    . *      *::  :          *. **    :                                    *
```

**Supplementary Figure S3: Sequence comparison of membrane-bound subunits of the ND3/NuoL/HycC/HyfB type.**

E._coli_NuoH	-MSWISPELIEILLTILKAVVILLVVVTCGAFMSFGERRLLGLFQNRYGPNRVGWGGSLQLVADMIKMFFKEDWIPKFSDRVIFTLAPMIAFTSLLLAFAIVPVSP-GWVVADLNIGILF	118

```
P._atrosepticum_HyfC      MTMQENPSLMMGIFALIQAGILLLLTP----LFTGISRQIRARMHSRRGP------GIWQDYRDIVKLLGRQDVAPAQSG-IIFRAMPWVLLGSMLLIAMTLPVLTRTSPFGGAG-DIIT	108
E._coli_HycD              ---------MSVLYPLIQALVLFAVAP----LLSGITRVARARLHNRRGP------GVLQEYRDIIKLLGRQSVGPDASG-WVFRLTPYVMVGVMLTIATALPVVTVGSPLPQLG-DLIT	99
                                   :  :  :::* ::: :.     :::   *   . ::.* **      *  *   *::*:: ::.  *  *.  :*   * : .  :*     :**      .   . .::

E._coli_NuoH              FLMMAGLAVYAVLFAGWSSNNKYSLLGAMRASAQTLSYEVFLGLSLMGVVAQAGSFNMTDIVNSQAHV-W-NVIPQFFGFITFAIAGVAVCHRHPFDQPEAEQELADGYHIEYSGMKFGL	236
P._atrosepticum_HyfC      LLYLFALFRFFFSLSGLDTGSTFAGIGASRELTLGVLVEPTLILGLLVVALIAGSTNIGTISATLTDA-WSSPTATLLALLACGFASFIEMGKIPFDVAEAEQELQEGPLTEYSGCALAL	227
E._coli_HycD              LLYLFAIARFFFAISGLDTGSPFTAIGASREAMLGVLVEPMLLLGLWVAAQVAGSTNISNITDTVYHWPLSQSIPLVLALCACAFATFIEMGKLPFDLAEAEQELQEGPLSEYSGSGFGV	219
                          :* : .:  : . ::* .:.. :: :** *     :  *  * *.*  ..  *** *:  *  :  .    .    .:.: : .:* .    : ***  ****** :*   ****  :.:

E._coli_NuoH              FFVGEYIGIVTISALMVTLFFGGW------QGPLLPPFIWFALKTAFFMMMFILIRASLPRPRYDQVMSFGWKICLPLTLINLLVTAAVILWQAQ	325
P._atrosepticum_HyfC      VKWGIGLKQVVVAALFLAVFMPFGKASEITAGSLLLALVLMLIKLLVIFVLASLFENSLARGRFLLTHRVTWVGFGVATLSFVFYLTGL------	316
E._coli_HycD              MKWGISLKQLVVLQMFVGVFIPWGQMETFTAGGLLLALVIAIVKLVVGVLVIALFENSMARLRLDITPRITWAGFGFAFLAFVSLLAA-------	307
                          .  *  :  :.:  ::: :*:          * **  ::   :*  . .::  *:. *: * *   .  . *       *  :   :.
```

**Supplementary Figure S4: Sequence comparison of membrane proteins of the ND1/NuoH/HycD/HyfC type.**

```
E._coli_NuoM              ------------MLLPWLILIPFIGG----FLCWQTERFGVKVPRWIA--LITMGLTLALSLQLWLQGGYSLTQSAGIPQWQSEFDMPWIPRFGI-SIHLAIDGLSLLMVVLTGLLGVLA	101
E._coli_NuoN              -------MTITPQNLIALLPLLIVGLTVVVVMLSIAW----RRNHFLNATLSVIGLNAALV-SLWFVGQ---AGA--------MDVTPL----------MRVDGFAMLYTGLVLLASLAT	87
P._atrosepticum_HyfD      MMESSSFMTNTALA-TLLL--PFIGA--LLTACSPKR----N-AKWLCTGFALLATLGTLV-LGWG---FLAHGK-------VAVTLPLLNYGNVALFGFTVDRISTLIVFAVVFLGLLV	99
P._atrosepticum_HyfF      -------MSDTSVLLLLLL--TPLGISVLNFSCHLAG----SAARRLVTTLHAIGIAALLVESLWVVSLIYYHGD-------ILAAHKW----------LHMDALSLLFLAILGIVSFIT	90
                                           *:    :*                   : :   :  :.    *    *                                  : :* :: *      : .. .

E._coli_NuoM              VLCSWKEIE--------------KYQGFFHLNLMWILGGVIGVFLAIDMFLFFFFWEMMLVPMYFLIAL-WGHKASDGKTRITAATKFFIYTQASGLVMLIAILALVFVHYNATGVW---	203
E._coli_NuoN              CTFAYPWLEGYNDN-------KDEFYLLVLI---AAL-GGILLANANHLASLFLGIELISLPLFGLVGYAFRQ-----KRSLEASIKYTILSAAASSFLLFGMA-LVYAQ---SGDLSFV	187
P._atrosepticum_HyfD      SLYSIGYLNTGN--REHPHEGTNRYYAFLLI---FIG-AMVGLVMSSTILGQLLFFEITGGCSWALIGY-YQT-----AKSQRSALKALLITHVA-SLGLFIAAAMLFIS---TG-----	198
P._atrosepticum_HyfF      GLYSIGYMRHEVDDGEVSVSGLCNYYGFFHL---FVF-TMLLTITSNNLIMMWAAVEATTLSSAFLVGT-YNQ-----RSSLEAAWKYIIICSVGVAFGLFGTI-LVYAN---ATNILAN	196
                             :   :.              .:  :. :         :    :  :       *        *:.  :            :: *  :   ..  . *:    :::     :

E._coli_NuoM              ----TFNYEELLNTPMSSGVEYLLML-GFFIAFAVKMPVVPLHGWLPDAHSQAPTAGSVD LAGILLKTAAYGLLRFSLPLFPNASAEFA----PIAMWLGVIGIFYGAWMAFAQTDIKRL	314
E._coli_NuoN              ALG------KNLGDGMLNEPLLLAGFGLMIVGLGFKLSLVPFHLWTPDVYQGAPAPVSTFLATASKIAIFGVVMRLF--LYA--PVGDSEAIRVVLAIIAFASIIFGNLMALSQTNIKRL	297
P._atrosepticum_HyfD      ----TFALSALSQ---LSGTPALLVFGGILFAAWGKSAQLPLQAWLPDAM-EAPTPVSAYLHAASMVKVGVYIYARA--IVESGHIPLV-IG-WVGIGMATLTIVYGFLMYLPQKDMKRL	306
P._atrosepticum_HyfF      PDDAIFWTDVLQHAALLDPVLMQLAFVFVLIGFGTKAGLFPMHAWLPDAHSEAPSPVSALLSAVLLNCALLIILRYN--IIINGTIGNR-FTSTLLIVFGLLSIAVAAFFILVQTDIKRL	313
                                           .       :  .:..   *   .*:: * **.   **:  *. *           :      :              :   :.   *  .  : : *.::***

E._coli_NuoM              IAYTSVSHMGFVLIAIYTG---SQLAYQGAVIQMIAHGLSAAGLFILCGQLYE---RIHTRDMRMMGGLWSKMKWLPALSLFFAVATLGMPGTGNFVGEFMILFGSFQVVPVITVIS--T	426
E._coli_NuoN              LGYSSISHLGYLLVALIALQT-GEMSMEAVGVYLAGYLFSSLGAFGVVSLMSSPYRGPDADSLFSYRGLFWHRPILAAVMTVMMLSLAGIPMTLGFIGKFYVLAVGVQAH-LWWLVGAVV	415
P._atrosepticum_HyfD      LAWSTITQLGYIFLALSIAIFGSREAFEGGIAYIFNHAFAKSLFFLVAGSLSY---CCGTRLLPRLRGVINKMPIVGIGFCIAALAIAGVPPLNGFFSKFPIFAAGFAMSHEFWVMTPLM	423
P._atrosepticum_HyfF      LAYSSVENMGLIAVALGIG---GPLGVLAAMLHTLNHSLGKTLLFCGSGNVLL---KYGTRDITVVKGMLNVVPFSAVLMTGGALALGGMPPFNIFLSEFMTVVAALSAD-HFWLTLVLL	426
                          :.:::: ::* : :*:      .  .  .       : :.    *   . :        :  :    *:               ::  *:*    *..:*  .  ..       :

E._coli_NuoM              FGLVFASVYSLAM-LHRAYFGKAKSQIASQELPGMSLRELFMILLLVVLLVLLGFYPQPILDTSHSAIGNIQQ-------------WFVNSVTTTRP-----------------	509
E._coli_NuoN              VGSAIGLYYYLRVAVSLYLHAPEQPGRDAPSNWQYSAGGI-VVLISALLVLVLGVWPQPLISIVRLAMPLM-------------------------------------------	485
P._atrosepticum_HyfD      VLVLIESVASFA--WLIYWFGQVVPGEPSEELVQASPLPI-TMRLVLLVLIVMSFCSS-VIAV----------------------IWLG-------------------------	486
P._atrosepticum_HyfF      LLLTLVM-GGLVRMISAIIFGSAPQQVSRGELGLLTTLPM-AILLVLMLIM-GTYIPHPVVQMLENAASVVLQRDSRAVQPHYTWPWASGSDNTVSTAGRRAVNTSSGEKSDLR	537
                          .   :     :        ..         .    :   :  : :  ::::        ::
```

**Supplementary Figure S5: Sequence comparison of membrane subunit of the MPS family proteins not found in FHL-1.**

```
E._coli_NuoK              -----MVSDRPSSTRLKPMLKSPTDIHVHSGWLYVCSPLAKKSTPIKA-------------VTSAARPTEPTPTI-AETFSDQR-PRENA-SRTKP------------------------	75
P._atrosepticum_HyfE      MTGSLIVNNLAGLMMFTSLMVIGVKRPTASCWLYSLQALILVLLFLTLSYTLDAHELRTWAISAFFTKVVLVPAILYFAFRTMRDPAHEGSVISLPVLMILAAAIVILCWFVAEPVQLPL	120
                               :*.:  .   :. ::   ..  . * ***  . *      :.              :::    .  .*:*   :*   * * .:.   : *

E._coli_NuoK              ----------INGKIMVKASMFIRASHLL----NRCSGSGD-DG-------------EVAAAAQGRYSLL----------------------PPRG	121
P._atrosepticum_HyfE      LATLKPALAVSLGHFMVGLLCIVTQRNILKQVFGYCLMENGSHLTLALLASKAPELVEIGIATDAIFAVIIMTILARKIWRTLNTLSVDQLTALKG	216

                                      *::**    ::   ::*    . *  ... .              *:. *::. ::::                        :*
```

**Supplementary Figure S6: Sequence comparison of the HyfK/NuoK membrane proteins not found in FHL-1.**

```
P._atrosepticum_HyfG      VLLIPHQERKVTYDNHVRAIQSDQKLGGNYLAQIRQKFPAAVLDEEWQTHDQLTITIKTESLPDVVEFIYYPLGGWLPVLFGNDERTLNGNYAVYYALSMEQGEKCWIVVKAYVDPLTLE	120
E._coli_HycE              --------------------MSEEKLGQHYLAALNEAFPGVVLDHAWQTKDQLTVTVKVNYLPEVVEFLYYKQGGWLSVLFGNDERKLNGHYAVYYVLSMEKGTKCWITVRVEVDANKPE	100
                                               *::*** :*** :.: **..***. ***:****:*:*.: **:****:**  **** ********.***:*****.****:* ****.*:. **  . *

P._atrosepticum_HyfG      FPSVTPRVPAAVWGEREIRDMYGLIPVGLPDERRLVLPDDWPDDLYPLRKDTMDYRLRPEPTTQIETYQFKNEGG-SEARIVPVGPLHITSDEPGHFRLFVDGERIVDADYRLFYVHRGM	239
E._coli_HycE              YPSVTPRVPAAVWGEREVRDMYGLIPVGLPDERRLVLPDDWPDELYPLRKDSMDYRQRPAPTTDAETYEFINELGDKKNNVVPIGPLHVTSDEPGHFRLFVDGENIIDADYRLFYVHRGM	220
                          :****************:*************************:*******:**** ** ***: ***:* ** * .: .:**:****:***************.*:*************

P._atrosepticum_HyfG      EKLAETRMGYNEVTFLSDRVCGICGFAHSVAYTSSVENALGIYVPPRAHAIRSILLEVERLHSHLLNIGLSSHFIGFDTGFMQFFRVREKSMALAELLTGARKTYGLNLIGGIRRDILKE	359
E._coli_HycE              EKLAETRMGYNEVTFLSDRVCGICGFAHSTAYTTSVENAMGIQVPERAQMIRAILLEVERLHSHLLNLGLACHFTGFDSGFMQFFRVRETSMKMAEILTGARKTYGLNLIGGIRRDLLKD	340
                          *****************************.***:*****:** ** **: **:**************:**:.** ***:**********.** :**:*******************:**:

P._atrosepticum_HyfG      QRQKGIKLVQEMRAEVTQLVDMLLNTPNMEQRTQGIGLLDRKIARDYSPVGPMIRGSGFARDMRFDHDYAGYSALPKTLFTLDGCDVFSRVMVRVQETLESLSMVEFGLDHLPEGPLLTE	479
E._coli_HycE              DMIQTRQLAQQMRREVQELVDVLLSTPNMEQRTVGIGRLDPEIARDFSNVGPMVRASGHARDTRADHPFVGYGLLPMEVHSEQGCDVISRLKVRINEVYTALNMIDYGLDNLPGGPLMVE	460
                          :  :  :*.*:** ** :***:**.******** *** ** :****:* ****:*.**.*** * ** :.**. **  :.: :****:**: **::*.  :*.*:::***:** ***:.*

P._atrosepticum_HyfG      GFTYRPYKFALGYTEAPRGEDVHWSMLGDNQKLFRWRCRAATYANWPVLRYMLRGNTISDAPLIIGSLDPCYSCTDRVTLVDIKKRKSTTVPYKEIERYSIERKNSPLK-	588
E._coli_HycE              GFTYIPHRFALGFAEAPRGDDIHWSMTGDNQKLYRWRCRAATYANWPTLRYMLRGNTVSDAPLIIGSLDPCYSCTDRMTVVDVRKKKSKVVPYKELERYSIERKNSPLK*	569
                          **** *::****::*****:*:**** ******:*************.*********:** *****************:*:**::*:**..*****:*************
```

**Supplementary Figure S7: Sequence comparison of hydrogenase-3 and hydrogenase-4 catalytic large subunits.**

The C-terminal peptide that is removed by proteolytic cleavage during enzyme maturation is shown in yellow. The Gly93-Glu94 location of the internal 10-His tag engineered on HyfG is shown in magenta.

```
E._coli_NuoC              --------------------------------MVNNMTDLTAQEPAWQTRDHLDDPVIGELRNRFGPDAFTVQATRTGVPVVWIKREQLLEVGDFLKKLPKPYVMLFDLHGMDERLRTHR	88
P._atrosepticum_HyfG      VLLIPHQERKVTYDNHVRAIQSDQKLGGNYLAQIRQKFPAAVLDEEWQTHDQLTITI---------------------------KTESLPDVVEFIY---------YPLGGWLPVLFGND	84
E._coli_HycE              --------------------MSEEKLGQHYLAALNEAFPGVVLDHAWQTKDQLTVTV---------------------------KVNYLPEVVEFLY---------YKQGGWLSVLFGND	64
                                                           :.:    .. :  ***:*:*   :                           * : * :* :*:          :   *    *  :

E._coli_NuoC              EGLPAADFSVFYHLISIDRN--RDIMLKVALAENDLHVPTFTKLFPNANWYERETWDLFGITFDGHPNLRRIMMPQTWKGHP--LRKDYPARATEFSPFELTKAKQDLEMEALTFKPEEW	204
P._atrosepticum_HyfG      ERTLNGNYAVYYALS-MEQGEKCWIVVKAYVDPLTLEFPSVTPRVPAAVWGEREIRDMYGLIPVGLPDERRLVLPDDWPDDLYPLRKDTMDYRL--RPE-------------PTTQIETY	188
E._coli_HycE              ERKLNGHYAVYYVLS-MEKGTKCWITVRVEVDANKPEYPSVTPRVPAAVWGEREVRDMYGLIPVGLPDERRLVLPDDWPDELYPLRKDSMDYRQ--RPA-------------PTTDAETY	168
                          *    ..::*:* *  :::.    * ::. :     . *:.*  .* * * ***  *::*:   * *: **:::*: * ..   ****         *               * . * :

E._coli_NuoC              GMKRGTENEDFMFLNLGPNHPSAHGAFRIVLQLDGEEIVDCVPDIGYHHRGAEKMGERQ-SWHSYIPYTDRIEYLGGCVNEMPYVLAVEKLAGITVPDRVNVIRVMLSELFRINSHLLYI	323
P._atrosepticum_HyfG      QFKNEGG-SEARIVPVGPLHITSDEPGHFRLFVDGERIVDADYRLFYVHRGMEKLAETRMGYNEVTFLSDRVCGICGFAHSVAYTSSVENALGIYVPPRAHAIRSILLEVERLHSHLLNI	307
E._coli_HycE              EFINELGDKKNNVVPIGPLHVTSDEPGHFRLFVDGENIIDADYRLFYVHRGMEKLAETRMGYNEVTFLSDRVCGICGFAHSTAYTTSVENAMGIQVPERAQMIRAILLEVERLHSHLLNL	288
                           : .    ..  .: :** * ::.   :: * :***.*:*.   : * *** **:.* : .::.    :**:  : * .:.  *. :**:  ** ** *.: ** :* *: *::**** :

E._coli_NuoC              STFIQDVGAMTPVFFAFTDRQKIYDLVEAITGFRMHPAWFRIGGVAHDLPRGWDRLLREFLDWMPKRLASYEKAALQNTILKGRSQGVAAYGAKEALEWGTTGAGLRATGIDFDVRKARP	443
P._atrosepticum_HyfG      GLSSHFIGFDTGFMQFFRVREKSMALAELLTGARKTYGLNLIGGIRRDILKEQRQKGIKLVQEMRAEVTQLVDMLLNTPNMEQRTQGIGLLDRKIARDYSPVGPMIRGSGFARDMRFDHD	427
E._coli_HycE              GLACHFTGFDSGFMQFFRVRETSMKMAEILTGARKTYGLNLIGGIRRDLLKDDMIQTRQLAQQMRREVQELVDVLLSTPNMEQRTVGIGRLDPEIARDFSNVGPMVRASGHARDTRADHP	408
                          .   :  *  : .:  *  *:.   :.* :** *   .   ***: :*: :       :: : *  .: .  .  *..  :: *: *:.  . : * ::. .*  :*.:*   * *  :
E._coli_NuoC              YSGYENFDFEIPVGGGVSDCYTRVMLKVEELRQSLRILEQCLNNMPEGPFKADHPLTTPP PKERTLQHIETLITHFLQVSWGPVMPANESFQMIEATKGINSYYLTSDGSTMSYRTRVRT	563
P._atrosepticum_HyfG      YAGYSALPKTLFTLDG-CDVFSRVMVRVQETLESLSMVEFGLDHLPEGPLLTEGFTYRPY---------------------------KFALGYTEAPRGEDVHWSMLGDNQKLFRWRCRA	519
E._coli_HycE              FVGYGLLPMEVHSEQG-CDVISRLKVRINEVYTALNMIDYGLDNLPGGPLMVEGFTYIPH---------------------------RFALGFAEAPRGDDIHWSMTGDNQKLYRWRCRA	500
                          : **  :   :    * .*  :*: ::::*   :* :::  *:::* **: .:     *                            . ::   ** :* : ::   ...   :* * *:

E._coli_NuoC              PSFAHLQQIPAAIRGSLVSDLIVYLGSIDFVMSDVDR--------------------------------	600
P._atrosepticum_HyfG      ATYANWPVLRYMLRGNTISDAPLIIGSLDPCYSCTDRVTLVDIKKRKSTTVPYKEIERYSIERKNSPLK	588
E._coli_HycE              ATYANWPTLRYMLRGNTVSDAPLIIGSLDPCYSCTDRMTVVDVRKKKSKVVPYKELERYSIERKNSPLK	568
                           ::*:   :   :**. :**  : :**:*   * .**
```

**Supplementary Figure S8: Sequence comparison of hydrogenase large subunits with NuoC from *E. coli* complex I.**

The C-terminal peptide that is removed by proteolytic cleavage during enzyme maturation is shown in yellow.

```
E._coli_NuoI              MTLKELLVGFGTQVRSIWMIGLHAFAKRETRMYPEEPVYLPPRYRGRIVLTRDPDGEERCVACNLCAVACPVGCISLQKAETKDGRWYPEFFRINFSRCIFCGLCEEACPTTAIQLTPDF	120
P._atrosepticum_HyfH      --------MMLKLFKTIWQAG------DVTVKYPFAPLEVCPGFRGKPE-----YDAQQCIACGACTIACPANALTMETDIETGA----RTWQLFLGRCIFCGRCEEVCPTRAIQLSADF	97
E._coli_HycF              ---------MFTFIKKVIKTG------TATSSYPLEPIAVDKNFRGKPE-----QNPQQCIGCAACVNACPSNALTVETDLATGE----LAWEFNLGHCIFCGRCEEVCPTAAIKLSQEY	96
                                   : . .:.:   *        *  **  *: :   :**:        . ::*:.*  *. *** ..::::.   ..       :.: :.:***** ***.*** **:*: ::

E._coli_NuoI              EMGEYKRQDLVYEKEDLLISG-----PGKYPEYNFYRMAGMAIDGKDKGEAENEAKPID-------VKSLLP--------------	180
P._atrosepticum_HyfH      ELAVTHKPDLYTRATFTLLKCRVCRQPFAAEKSVEYAMALLAHSGIDAESIEEMRPQFETCPACKREQSLNHHGRTNLSYHIGEAK	183
E._coli_HycF              ELAVWKKEDFLQQSRFALCNCRVCNRPFAVQKEIDYAIALLKHNGDS--RAENHRESFETCPECKRQKCLVPSDRIELTRHMKEAI	180
                          *:.  :: *:  .    * .      *    :   * :* :  .* .    *:    ::        :.*
```

**Supplementary Figure S9: Sequence comparison of electron transferring subunits of the HycF/HyfH/NuoI type.**

```
P._atrosepticum_HyfI      MS-QPEMKINHHVSQPITLDEQAAQLKGRLLKDIQRSAYVYRVDCGGCNGCEIEIFAAITPLFDAERFGIKVVASPRHADILLFTGAVTRAMRVPALRAYESAPDPKICISYGACGCGGG	119
E._coli_HycG              MSNLLGPRDANGIPVPMTVDESIASMKASLLKKIKRSAYVYRVDCGGCNGCEIEIFATLSPLFDAERFGIKVVPSPRHADILLFTGAVTRAMRSPALRAWQSAPDPKICISYGACGNSGG	120
                          **     :  : :  *:*:**. *.:*. ***.*:**********************:::************* ******************* *****::*************** .**

P._atrosepticum_HyfI      IFHDLYCVWGGSESIVPIDVWIPGCPPTPAATIHGFAVALGLLEQKLKGQDHIQLPGERAETLLPTIPLATRVMVEREARRLAGYRKGREISDRLLTLLAGSDLSTAAKKLDVWLSEEND	239
E._coli_HycG              IFHDLYCVWGGTDKIVPVDVYIPGCPPTPAATLYGFAMALGLLEQKIHARGPGELDEQPAEILHGDMVQPLRVKVDREARRLAGYRYGRQIADDYLTQLGQ-----GEEQVARWLEAEND	235
                          ***********::.***:**:***********::***:********::.:.  :*  : ** *   :    ** *:********** **:*:*  ** *.      . :::  **. ***

P._atrosepticum_HyfI      PRLREIVQCLTQLLREAPNV*	259
E._coli_HycG              PRLNEIVSHLNHVVEEARIR-	255
                          ***.***. *.:::.**
```

**Supplementary Figure S10: Sequence comparison of hydrogenase small subunits from formate hydrogenlyases.**

The small subunit of *E. coli* Hydrogenase-3 is encoded by the *hycG* gene, while the small subunit of *P. atrosepticum* Hydrogenase-4 is encoded by *hyfI*.
